# CD276 immature glycosylation drives colorectal cancer aggressiveness and T cell mediated immune escape

**DOI:** 10.1186/s12964-026-02672-y

**Published:** 2026-01-20

**Authors:** Janine Soares, Dylan Ferreira, Andreia Miranda, Martina Gonçalves, Marta Relvas-Santos, Andreia Brandão, Paula Paulo, Sofia Cotton, Rui Freitas, Mariana Magalhães, Eduardo Ferreira, Beatriz Marinho-Santos, Luís Pedro Afonso, André M. N. Silva, Carlos Palmeira, Francisco Amado, Andreia Peixoto, Lúcio Lara Santos, José Alexandre Ferreira

**Affiliations:** 1 Research Center of IPO-Porto (CI-IPOP)/RISE@CI-IPOP (Health Research Network), Portuguese Oncology Institute of Porto (IPO-Porto)/Porto Comprehensive Cancer Center (P.Ccc) Raquel Seruca, Porto, Portugal; 2 School of Medicine and Biomedical Sciences (ICBAS), University of Porto, Porto, Portugal; 3 LAQV-REQUIMTE, Department of Chemistry, University of Aveiro, Campus Universitário de Santiago, Aveiro, Portugal; 4 i3S – Instituto de Investigação e Inovação em Saúde, Universidade Do Porto, Porto, Portugal; 5 LAQV-REQUIMTE, Department of Chemistry and Biochemistry, Faculty of Sciences, University of Porto, Porto, Portugal; 6 ESS, Polytechnic of Porto, Porto, Portugal; 7 Pathology Department, Portuguese Oncology Institute of Porto, Porto, Portugal; 8 Immunology Department, Portuguese Oncology Institute of Porto, Porto, Portugal; 9 School of Medicine, Biomedical Sciences of University Fernando Pessoa, Porto, Portugal; 10 Department of Surgical Oncology, Portuguese Oncology Institute of Porto, Porto, Portugal; 11 Experimental Pathology and Therapeutics group, Research Centre, Portuguese Oncology Institute of Porto, R. Dr. António Bernardino de Almeida, Porto, 4200-072 Portugal

**Keywords:** Colorectal cancer, Cancer metastasis, Glycoproteomics, CD276, Immune suppression

## Abstract

**Background:**

Colorectal cancer (CRC) progression is fuelled by immune evasion, yet the underlying molecular mechanisms remain to be fully characterized. CD276 (B7-H3), an immune checkpoint glycoprotein frequently overexpressed in aggressive tumors, is extensively modified by glycosylation, a process known to regulate protein stability, localization, and immune interactions. However, its glycosylation-dependent functions in CRC remain unclear.

**Methods:**

TCGA Transcriptomic data were analysed to identify glycogene alterations linked to patient prognosis. The O-glycome of advanced CRC and normal mucosa was profiled by mass spectrometry. CD276 expression and glycosylation were examined in primary tumors, lymph nodes, and metastases by immunohistochemistry, proximity ligation assays, and dual immunofluorescence. CRC proteomic datasets from PRIDE (≥ 90 cases) were reanalyzed to map CD276 immature glycosylation across differentiation states. *C1GALT1* knockout CRC cell lines were generated with CRISPR-Cas9 to mimic immature glycosylation in tumors, and CD276 was silenced with siRNAs. Immunoprecipitation, lectin blotting, protein stability assays, proliferation, invasion, phosphoproteomics, and T cell co-culture experiments were used to assess functional consequences.

**Results:**

Downregulation of *B3GNT6* and *C1GALT1* or *C1GALT1C1* defined an immature O-glycosylation phenotype associated with poor prognosis. Glycomic profiling revealed Tn- and sialyl-Tn(sTn)-enriched glycophenotypes in both epithelial- and mesenchymal-like tumors, with subtype-specific patterns. CD276 colocalized with Tn and sTn, carried immature O-glycans absent from healthy tissues, and was enriched in right-sided and metastatic CRC, correlating with worse survival. PRIDE reanalysis suggested widespread CD276 expression and revealed differentiation-linked glycosylation, which was denser in the IgV and IgC domains of epithelial-like tumors and sparser, membrane-proximal in mesenchymal-like tumors. *C1GALT1* knockout in CRC cells enhanced invasion while increasing CD276 stability and transcription, driving its overexpression. Aberrantly glycosylated CD276 promoted proliferation, invasion, kinase-driven signalling, and T cell suppression while driving cytokines toward immunosuppression. TCGA confirmed that high *CD276* and low *C1GALT1* expression correlated with transcriptional signatures of heightened immune checkpoint activity and T cell exhaustion.

**Conclusions:**

Immature CD276 glycosylation promotes CRC aggressiveness and immune escape, representing a candidate prognostic biomarker and therapeutic target.

**Graphical Abstract:**

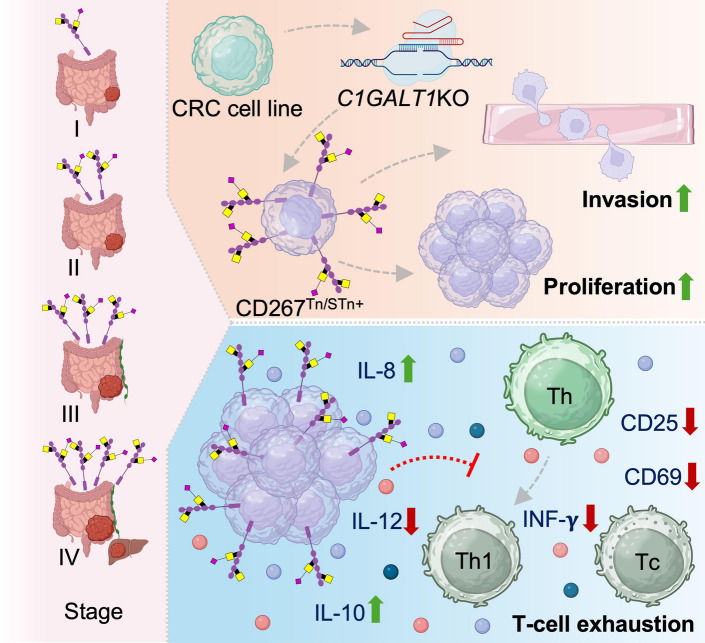

Colorectal cancer (CRC) progression is closely linked to immune evasion, yet the molecular mechanisms underlying this process remain poorly understood. This study identifies CD276 (B7-H3) as a glycosylation-driven regulator of CRC aggressiveness and demonstrates how *O*-glycosylation remodelling promotes tumor immune escape. These findings establish CD276 as a potential therapeutic target and highlight the role of glycoproteoform-specific immune modulation in cancer progression.

**Supplementary Information:**

The online version contains supplementary material available at 10.1186/s12964-026-02672-y.

## Background

Colorectal cancer (CRC) remains one of the most lethal malignancies worldwide, with poor survival rates for patients with metastatic disease [1]. While immune checkpoint inhibitors (ICIs) have transformed into cancer therapies, their efficacy is largely restricted to tumors with microsatellite instability (MSI) and a high tumor mutational burden (TMB) [2]. Most CRC cases, particularly those with microsatellite stability (MSS), which represent approximately 95% of metastatic CRCs overall, remain resistant [3], highlighting a critical gap in the understanding of alternative immune evasion mechanisms that drive tumor progression.

Among the emerging immune modulators in cancer, CD276 (B7-H3) has gained attention as a noncanonical immune checkpoint molecule with a pivotal role in tumor progression. CD276 is a type I transmembrane glycoprotein belonging to the B7 family of costimulatory and coinhibitory immune regulators, yet its physiological immune function remains controversial [4, 5]. Unlike classical immune checkpoints such as PD-L1 and CTLA-4, CD276 lacks a well-defined receptor and can perform both immunosuppressive and pro-tumorigenic functions depending on the tumor context [5–7]. Nonetheless, CD276 overexpression has been reported across multiple malignancies, including lung [8], breast [9], prostate [10], and colorectal [11] cancers, where it is correlated with poor prognosis, increased metastatic potential, and therapy resistance. In addition to being involved in immune regulation, CD276 has been implicated in epithelial‒mesenchymal transition (EMT) [12], resistance to apoptosis [13], and angiogenesis [14]. Nevertheless, the molecular mechanisms governing its function remain incompletely understood, particularly at the posttranslational level.

Glycosylation plays a crucial role in regulating immune checkpoint function and the response to immunotherapy, including that of CD276 [15, 16]. Recent evidence indicates that core fucose *N*-glycosylation, which is mediated by FUT8, stabilizes CD276, modulates its surface expression, and suppresses T cell activation and proliferation in triple-negative breast cancer [15]. Inhibiting core fucosylation has emerged as a promising strategy to enhance antitumor immune responses, underscoring the importance of glycosylation as a therapeutic target. However, the role of *O*-glycosylation, an essential posttranslational modification in immune regulation and tumor progression [17], in CD276 function remains poorly defined. In CRC, extensive remodelling of the cancer glycocalyx leads to an immature *O*-glycosylation phenotype, primarily due to Cosmc (encoded by *C1GALT1C1*) dysregulation [18, 19]. Cosmc is essential for the function of Core 1 β1,3-galactosyltransferase 1 (encoded by *C1GALT1*), the key enzyme responsible for *O*-glycan elongation via Core 1 synthesis. Loss of Cosmc activity results in the accumulation of the Tn antigen, a hallmark of aberrant *O*-glycosylation [20]. These alterations are predominantly driven by *C1GALT1C1* promoter hypermethylation and, in some cases, loss-of-function mutations [21, 22]. This dysregulation has been strongly linked to cancer invasion, mainly by disrupting normal *O*-glycosylation of cell surface proteins, leading to impaired cell‒cell adhesion, enhanced migration, and metastasis [23–25], while also promoting immune evasion via macrophage-binding lectins on innate immune cells [26]. While the role of C1GalT1-specific chaperone 1 in CRC is well established, the direct contribution of Core 1 β1,3-galactosyltransferase 1 itself to tumor progression remains poorly understood [18]. More broadly, despite growing evidence of the impact of *O*-glycosylation on cancer biology and immune modulation, how it shapes CD276 function, particularly in the context of immune interactions and tumor progression, remains largely unknown.

In this study, through a systematic analysis of the cancer glycome, we identified CD276 as a metastasis-associated glycoprotein that is extensively modified by *O*-glycosylation in CRC. We demonstrate that tumors with poor prognostic features exhibit aberrant glycogene expression, leading to an immature *O*-glycosylation phenotype that enhances malignancy. Using *C1GALT1* knockout CRC cells (*C1GALT1*KOs), which recapitulate the cancer-associated glycocalyx, we revealed that *O*-glycan remodelling enhances CD276-driven tumor invasion and impairs T cell activation. These findings establish CD276 as a glycosylation-dependent immune checkpoint and highlight its potential as a therapeutic target to overcome immune evasion in CRC.

## Materials and methods

### Clinical tissue samples

A retrospective cohort of 40 formalin-fixed paraffin-embedded (FFPE) CRC tissue samples was selected from the institutional pathology biobank of the Portuguese Institute of Oncology of Porto (IPO Porto). Samples were collected from patients who underwent surgical resection for colorectal (adeno)carcinomas between 2005 and 2012. The cohort included 18 female and 22 male patients aged 28–76 years (mean ± SD: 61 ± 11 years). Whenever present, adjacent histologically normal mucosa was also included for comparative analysis. In addition to CRC samples, a reference panel of healthy tissues, including liver, stomach, pancreas, appendix, lung, testis, thyroid, and gallbladder, was analysed. Tumor and healthy tissue sections were further screened for CD276 and aberrant glycosylation markers (Tn and sTn antigens) via multiple immunoassays: immunohistochemistry (IHC), Proximity Ligation Assay (PLA), and dual immunofluorescence staining with lectins and specific antibodies. Clinical and pathological data were retrieved from patient medical records and are summarized in Table 1 The antibodies and experimental conditions are detailed in Table S5.

**Table 1:** Clinicopathological data associated with the tumor tissues assessed in this study (*n* = 40)

	***n*** (%)
Stage	
I	1 (2.5)
II	4 (10.0)
III	10 (25.0)
IV	25 (62.5)
Tumor (T)	
T1	1 (2.5)
T2	1 (2.5)
T3	31 (77.5)
T4	6 (15.0)
Missing Information	1 (2.5)
Lymph node metastases (N)	
N0	11 (27.5)
N1	12 (30.0)
N2	14 (35.0)
N3	3 (7.5)
Tumor Location	
Right colon	7 (17.5)
Left colon	23 (57.5)
Rectum	10 (25.0)

### Transcriptomic analysis of the TCGA-COADREAD cohort

RNA sequencing (RNA-seq) data for CRC were obtained from The Cancer Genome Atlas (TCGA) COADREAD cohort, comprising 623 tumor tissues and 51 matched normal adjacent tissues. Processed gene expression data (log2(FPKM + 1)) and associated clinical annotations, including age, sex, tumor stage, histological subtype, and overall survival, were downloaded via the UCSC Xena platform (https://xena.ucsc.edu/), which hosts curated TCGA datasets. After excluding samples with incomplete clinical data, a final cohort of 598 tumor samples and 51 normal adjacent tissue samples was included for analysis. The clinical characteristics of the COADREAD patients are summarized in Table 2.

**Table 2:** Clinicopathological characteristics of CRC patients included in the TCGA COADREAD dataset

	n	n (%)
Age	598	66.34 12.78 (Mean±SD)
Gender	598	
Female		280 (47)
Male		318 (53)
Histological type	587	
Colon Adenocarcinoma		376 (64)
Colon Mucinous Adenocarcinoma		62 (11)
Rectal Adenocarcinoma		137 (23)
Rectal Mucinous Adenocarcinoma		12 (2)
Clinical stage	598	
I		104 (17)
II		227 (38)
III		179 (30)
IV		88 (15)
Vital status	598	
Alive		479 (80)
Dead		119 (20)

### Proteomic revisitation of the TCGA-COADREAD cohort for CD276 glycosylation

A subset of 95 colorectal cancer (COADREAD) cases from TCGA, analysed via mass spectrometry and annotated with clinicopathological data in Zhang et al*.* [27], was retrieved from the PRIDE Archive (dataset identifier PXD002080). The proteomics data were reanalyzed to identify and quantify glycoproteins modified with Tn and sialyl-Tn (sTn) antigens. The samples were stratified into epithelial-like and mesenchymal-like subtypes according to the TCGA transcriptomic classification used by Zhang et al*.* [27]. Further classification was based on the presence of unglycosylated CD276 or CD276 glycosylated with Tn/sTn antigens (CD276-Tn/sTn). All data processing and visualization were performed in R version 4.2.3. Data wrangling and statistical plotting were conducted using the tidyverse [28] and ggpubr packages. CD276-Tn/sTn–positive samples were manually curated to identify candidate glycosylation sites specific to epithelial- and mesenchymal-like subtypes. These glycosites were visualized as a heatmap via the pheatmap package, enabling subgroup clustering and the identification of potential molecular patterns.

### Cell lines and culture conditions

Human CRC cell lines SW480 (RRID:CVCL_0546), SW620 (RRID:CVCL_0547), HCT116 (RRID:CVCL_0291), RKO (RRID:CVCL_0504), LS174T (RRID:CVCL_1384), and HCA7 (RRID:CVCL_0289) were obtained from the American Type Culture Collection (ATCC, Manassas, VA, USA). Cells were cultured in RPMI 1640 GlutaMAX™ medium (Gibco) supplemented with 10% fetal bovine serum (FBS) and 1% penicillin–streptomycin at 37 °C in a humidified incubator with 5% CO₂. Cells were screened weekly and confirmed to be mycoplasma free throughout the study.

### CRISPR-Cas9 glycoengineered cell models

Two guide RNAs targeting the human *C1GALT1* gene (sequences: *GTAAAGCAGGGCTACATGAG* and *ACAACACTTTGTTACAACGC*) were cloned and inserted into a dual-guide RNA CRISPR-Cas9 expression vector (pRP[2CRISPR]-Puro-hCas; VectorBuilder). Wild-type SW480 and SW620 CRC cell lines were transfected with the CRISPR‒Cas9 plasmid (1 µg) via jetPRIME® transfection reagent (Polyplus) according to the manufacturer’s protocol. Single-cell clones were isolated by limiting dilution in 96-well plates, and successful *C1GALT1* KO clones were identified via Indel detection by amplicon analysis (IDAA) via the ABI PRISM™ 3010 Genetic Analyser (Thermo Fisher Scientific) and confirmed via Sanger sequencing. Three independent KO clones with distinct out-of-frame indels were selected for further studies. In addition, clones harboring silent mutations were used as phenotypic control cell lines. The IDA results were analysed using the Peak Scanner Software v1.0 (Thermo Fisher Scientific).

### siRNA-mediated CD276 silencing

*CD276* gene silencing was performed in SW620 glycoengineered colorectal cancer cell models via reverse transfection with siRNA. Two Silencer® Select siRNAs targeting distinct exons of human CD276 (s37289, exon 10; and s37288, exon 7; Invitrogen) were used to ensure target specificity. A Silencer® Select negative control siRNA (catalogue no. 4390843; Invitrogen) was included in all the experiments as a nontargeting control, as previously described [29]. Cells were detached and seeded into 24-well plates at a density of 1 × 105 cells/well prior to transfection. siRNAs and Lipofectamine® RNAiMAX (Invitrogen) were separately diluted in Opti-MEM® Reduced Serum Medium (Gibco), incubated for 5 min at room temperature, and combined to form siRNA–lipid complexes. Cells were then incubated with the complexes for 72 h at 37 °C. Each condition was plated in duplicate wells per experiment, and all experiments were performed in at least three independent biological replicates. To evaluate gene silencing efficiency, *CD276* mRNA expression was quantified via RT‒qPCR with the TaqMan® Gene Expression Assay Hs00987207_m1 (Thermo Fisher Scientific). *GAPDH* and *ACTB* were used as endogenous controls. To confirm the specificity and rule out off-target effects, both nontargeting negative control siRNA and mock-transfected cells (no siRNA, Lipofectamine only) were included as negative controls. Effective knockdown was confirmed only when both *CD276*-targeting siRNAs produced consistent reductions in mRNA levels compared to controls. Cell viability and morphology were also monitored microscopically to exclude transfection-related cytotoxicity.

### Proliferation assay

Cell proliferation was assessed using the Cell Proliferation BrdU ELISA (colorimetric) kit (Roche), according to the manufacturer’s instructions. Absorbance was measured at 450 nm in a microplate reader. Each assay was performed in triplicate, with three technical replicates per condition.

### Invasion assay

Invasion potential was evaluated using Falcon® Permeable Supports for 24-well plates with 8.0 µm transparent PET membranes (Corning) coated with Matrigel® Basement Membrane Matrix (50 µg/mL, Corning). Inserts were pre-coated and incubated at 37 °C for 1 h prior to cell seeding. Sub-confluent cells were detached using trypsin/EDTA (Thermo Fisher Scientific) and seeded in the upper chamber at a density of 5 × 104 cells/mL. After 24 h of incubation at 37 °C, non-invading- cells were removed from the upper surface, and the inserts were washed with PBS and fixed in 4% paraformaldehyde (Sigma-Aldrich). Filters were mounted in Vectashield with DAPI (Vector Laboratories) and imaged using a Zeiss Axiovert 200 M fluorescence microscope (Carl Zeiss). Each assay was performed in triplicate, with five technical replicates per condition. Results were normalized to cell proliferation and expressed as the fold change relative to the control.

### RT‒qPCR for CD276 and glycogene expression

Total RNA was extracted via TriPure Isolation Reagent (Roche Diagnostics). cDNA synthesis and mRNA quantification were performed as previously described [30]. Quantitative RT‒PCR was carried out using TaqMan Gene Expression Assays for CD276 (Hs00987207_m1) and B3GNT6 (Hs00371066_s1) on a 7500 Fast Real-Time PCR System (Applied Biosystems). GAPDH (Hs03929097_g1) and ACTB (Hs99999903_m1) were used as endogenous controls. All samples were analysed in duplicate, and relative gene expression was calculated with the 2^–ΔCt method.

### Western blot for CD276 and glycan detection

Total and plasma membrane protein extracts, as well as CD276-immunoprecipitated fractions, were analysed by Western blotting. Total protein lysates were prepared in Tris–HCl (25 mM, pH 7.4), NaCl (150 mM), MgCl₂ (5 mM), 1% NP-40, and 5% glycerol supplemented with Halt™ Protease and Phosphatase Inhibitor Cocktail (Thermo Fisher Scientific). Plasma membrane proteins were isolated by ultracentrifugation, as described by Fernandes et al. [31]. Proteins were resolved by SDS‒PAGE (4–20% precast gels, Bio-Rad) and transferred to nitrocellulose membranes (Cytiva). The membranes were probed with anti-CD276 antibodies and biotinylated Vicia villosa agglutinin (VVA) lectin (Vector Laboratories) to detect GalNAc residues associated with the Tn antigen. Lectin binding was visualized with streptavidin-HRP and chemiluminescent detection. The results were normalized to the total protein content determined by Ponceau S staining. Antibodies, lectins, and experimental conditions are detailed in Table S5.

### CD276 immunoprecipitation

CD276 was immunoprecipitated from plasma membrane protein extracts using Pierce™ Protein G agarose beads (Thermo Fisher Scientific). Beads were pre-blocked with 1% BSA (Sigma‒Aldrich) for 1 h at 4 °C. Protein extracts were precleared with BSA-blocked beads for 2 h at 4 °C to reduce nonspecific binding. Supernatants were incubated with a CD276 polyclonal antibody (10 µg) at 4 °C for 2 h, followed by an overnight incubation with freshly blocked beads. After washing, the immune complexes were eluted in SDS loading buffer at 95 °C. Eluted proteins were resolved on 4–20% gradient SDS‒PAGE gels (Bio-Rad) and transferred to nitrocellulose membranes (Cytiva). Immunodetection was performed with an anti-CD276 antibody and VVA lectin, as detailed in Table S5.

### Flow cytometry for CD276 and glycan detection

Flow cytometry was used to assess the expression of Tn, sTn, T, and sT antigens, as well as that of CD276, as previously described by Peixoto et al. [32]. Detailed antibody information and staining conditions are listed in Table S5. To validate sialylated glycans specificity, isotype controls and cells treated with 70 mU of α-neuraminidase (*Clostridium perfringens*, Sigma‒Aldrich) were used as negative controls. Data were acquired on a Beckman Coulter FC500 flow cytometer and analysed using CXP software (Beckman Coulter).

### Protein stability assay using cycloheximide chase (CHX)

To assess CD276 protein stability, 1 × 10⁶ SW480 and SW620 colorectal carcinoma cells were seeded per well in 6-well plates and cultured overnight in complete RPMI 1640 GlutaMAX™ medium (Gibco). The cells were treated with CHX (20 μM, Sigma‒Aldrich) dissolved in DMSO to inhibit de novo protein synthesis. Control wells were treated with an equivalent volume of DMSO. Whole-cell protein extracts were collected over time for 24 h post-treatment in RIPA buffer (50 mM Tris–HCl, pH 8.0; 150 mM NaCl; 1% NP-40; 0.5% sodium deoxycholate; 0.1% SDS) freshly supplemented with Halt™ Protease and Phosphatase Inhibitor Cocktail (Thermo Fisher Scientific). For each time point, 10 µg of protein was resolved via SDS‒PAGE on 4–20% precast gradient gels (Bio‒Rad) and transferred onto nitrocellulose membranes (Cytiva). Protein transfer and equal loading were verified by staining with Ponceau S solution (Sigma‒Aldrich). The membranes were blocked and immunoblotted with primary antibodies against CD276 and appropriate HRP-conjugated secondary antibodies, as detailed in Table S5.

### *O*-glycomics of cell models and patient samples

*O*-glycome characterization of CRC and matched healthy tissues was performed on 10 µm FFPE sections. *O*-glycans were released after *N*-glycan extraction as described by Relvas-Santos et al. [33]. Specifically, on-tissue reductive β-elimination (1 M NaBH₄ in 50 mM KOH, overnight at 50 °C) was employed for *O*-glycan release, followed by desalting using cation-exchange resin (AG 50 W-X8; Bio-Rad). For CRC cell lines, *O*-glycans were profiled via the Cellular *O*-Glycome Reporter/Amplification (CORA) approach [34], following the protocol of Fernandes et al*.* [31]. The released glycans were dried and permethylated as previously described [33]. NanoLC‒MS/MS analysis was performed in a Ultimate 3000 nano-LC system coupled to a Q Exactive mass spectrometer with an EASY-Spray nano-electrospray source (Thermo Fisher Scientific). Eluent A was 0.2% formic acid in water; eluent B was 0.2% formic acid in acetonitrile. The samples were loaded onto a PepMap C18 trap column (5 µm, Thermo Fisher Scientific) and washed isocratically (90% A, 10% B, 30 µL/min). After 3 min, the mixture was redirected to an analytical column (PepMap C18, 100 Å, 150 mm × 75 µm, 3 µm particle size) operated at 0.3 µL/min and 35 °C. Glycan separation was achieved with a linear gradient: 10% B at 10 min, 38% B at 20 min, 50% B at 55 min, and 90% B at 65 min. The column was held at 90% B for 10 min before re-equilibration. Mass spectrometry (MS) was performed in positive ion mode with a scan range of *m/z* 400–2000 for analysis of the cell lines and *m/z* 280–2000 for analysis of *O*-glycans extracted from tissues, 120,000 resolution (Full MS), spray voltage of 1.9 kV, and capillary temperature of 275 °C. Data-dependent MS/MS (Top 15) was acquired at 30,000 resolution using higher-energy collisional dissociation (HCD, 30% normalized collision energy, 4.0 m*/z* isolation window), with a 45 s dynamic exclusion. Data analysis was performed with Xcalibur v3.0 (Thermo Fisher Scientific), applying Gaussian smoothing, 10 ppm mass tolerance, four-decimal mass precision, and default baseline subtraction. Glycan structures were assigned considering retention time, monoisotopic m/z, and MS/MS spectra. The relative abundance resulted from the sum of the extracted ion chromatogram areas for each glycan structure in relation to the sum of the chromatographic areas of all identified glycans. Isomeric and isobaric structures were not differentiated. Glycan representations were created with GlycoWorkBench v2.1.

### Phosphoproteomics of cell models

Glycoengineered SW620 cells, with or without CD276 knockdown by siRNA, were harvested for whole protein extraction by cell scrapping inn lysis buffer (1% sodium deoxycholate, 10 mM tris(2-carboxyethyl)phosphine hydrochloride), 40 mM chloroacetamide, and 100 mM Tris) supplemented with Halt™ Protease and Phosphatase Inhibitor Cocktail (1x) (Thermo Fisher Scientific). Lysates were heated at 95 °C and sonicated in an ultrasound bath. After protein quantification, 200 μg of total protein was digested overnight at 37 °C using trypsin (Promega; 1 μg per 50 μg of protein) in 50 mM ammonium bicarbonate. Sodium deoxycholate was precipitated with 2% formic acid by centrifugation. Dried samples were desalted using Pierce™ Peptide Desalting Spin Columns (Thermo Fisher Scientific) and enriched for phosphopeptides with TiO₂-based spin columns (High-Select™ TiO2 Phosphopeptide Enrichment Kit, Thermo Fisher Scientific) according to the manufacturers’ protocol. Samples were analysed in a nanoLC-nESI-MS/MS on a Q Exactive™ mass spectrometer (Thermo Fisher Scientific) coupled to a Vanquish neoUHPLC nano-LC system. Eluent A was aqueous formic acid (0.1%), and eluent B was formic acid (0.1%) in 80% acetonitrile. The samples were injected directly into a trapping column (PEPMAP NEO C18, 5 μm particle size 300 μm × 5 mm) and separated on an analytical column (EASY-Spray C18 PepMap, 100 Å, 150 mm × 75 μm ID and 3 μm particle size) at a flow rate of 0.20 μL/min. The column temperature was set at 35 °C. Separation was performed via a multistep linear gradient to obtain 9% eluent B at 12 min, 36% eluent B at 102 min, and 99% eluent B at 107 min. The column was maintained at 99% eluent B for 6 min before re-equilibration at 2.5% eluent B. The mass spectrometer was operated in positive ion mode, with a *m/z* range from 375–1600 with 70 k resolution (Full MS), a spray voltage of 1.9 kV, and a transfer capillary temperature of 275 °C. Tandem MS (MS/MS) data were acquired using a data-dependent method with a dynamic exclusion of 16.0 s at a 17,500 resolution. The top 10 most intense ions were selected for higher energy collisional dissociation (HCD) using 28% normalized collision energy (nce) and an isolation window of 1.4 m/z. Raw data were processed using MaxQuant v2.4.7.0 with the reviewed human proteome database from UniProt (20,408 entries; accessed on 2023/07/07). In addition to the default settings for the Orbitrap instrument and label-free quantification, the following settings were applied: trypsin/P as the enzyme, three missed cleavages, fixed carbamidomethyl cysteine modification, and the variable modifications methionine oxidation, protein *N*-terminal acetylation, and serine/threonine/tyrosine phosphorylation, and match between runs enabled. Using the Perseus framework v2.0.11.0, only reverse phosphoproteins identified by site were included, while contaminants were excluded. Only phosphoproteins with a minimum of three valid values in at least one group were considered. Missing values were imputed based on a normal distribution. Phosphomatics was subsequently used for phosphosite mapping, functional annotation, and visualization of kinase–substrate networks. Differentially phosphorylated proteins were functionally annotated using Kyoto Encyclopedia of Genes and Genomes (KEGG) and Reactome pathway enrichment, and kinase–substrate relationships were inferred via kinase–substrate enrichment analysis (KSEA).

### T cell activation and proliferation assays

Human peripheral T cells were isolated from buffy coats of healthy donors. T cells were enriched using the RosetteSep™ Human T cell Enrichment Cocktail (STEMCELL Technologies) following the manufacturer’s protocol. Briefly, buffy coats were centrifuged at 1200 × g for 30 min at room temperature (acceleration 5, no brake) to isolate peripheral blood mononuclear cells (PBMCs). PBMCs were incubated with RosetteSep reagent, diluted 1:1 in PBS containing 2% fetal bovine serum (FBS), and layered over Histopaque-1077 (Sigma‒Aldrich) for density gradient centrifugation (30 min, 1200 × g, RT, no acceleration or brake). The T cell–enriched layer was collected and washed three times with PBS. Isolated T cells were cultured in RPMI 1640 GlutaMAX™ medium supplemented with 10% FBS, 1% penicillin/streptomycin, and 100 U/mL interleukin-2 (PeproTech, 200–02) and activated for 3 days with plate-bound anti-CD3 and anti-CD28 antibodies (Table S5). For proliferation tracking, T cells were labelled with CellTrace™ and the carboxyfluorescein succinimidyl ester (CFSE) dye (Thermo Fisher Scientific). Cells were resuspended in PBS and incubated with 2.5 μM CFSE for 8min at 37 °C. Labelling was quenched by adding complete medium, and the cells were washed and resuspended for coculture. Labelled T cells were then co-cultured with glycoengineered SW620 cells (with or without *CD276* knockdown) at a tumor-to-T cell ratio of 1:5 in 24-well plates for 5 days at 37 °C and 5% CO₂. At the endpoint, the conditioned media were collected for cytokine analysis, and the cells were harvested by incubation with cold PBS on ice for 20 min. After being washed, the cells were stained with Fixable Viability Stain 780 for 15 min at 4 °C in the dark, followed by surface marker staining for CD3, CD4, CD8, CD25, and CD69 (Table S5) for 40 min at 4 °C. Flow cytometry analysis was performed on a NAVIOS flow cytometer (Beckman Coulter). Data were analysed with the Kaluza C software (v1.2) to assess viability, activation marker expression, mean fluorescence intensity (MFI), and the percentage of positive cells across relevant T cell subsets.

### Cytokine evaluation

Conditioned media from CRC cell–T cell co-cultures were collected after 120 h of direct contact. IL-1β, IL-2, IL-4, IL-5, IL-6, IL-8, IL-10, IL-12 (p70), IL-13, IL-17A, IL-23, IFN-γ, TNF-α, and GM-CSF levels were quantified by a Luminex-based human multiplex cytokine assay, which was outsourced to EVE Technologies, according to standard protocols.

### Tissue-level analysis of CD276 and glycan levels via IHC

FFPE sections of colorectal tumors and healthy tissues were analysed by IHC for CD276, Tn, and sTn antigens, as previously described by Peixoto et al*.* [32]. The antibodies, lectins, and detection reagents used are listed in Table S5. CD276 and anti-sTn antibodies were detected with the the Novolink™ Polymer Detection System (Leica Biosystems) according to the manufacturer’s instructions. For Tn antigen detection, biotinylated VVA was used, followed by streptavidin-horseradish peroxidase (HRP) conjugation (Thermo Fisher Scientific, ready-to-use) and chromogenic development with ImmPACT® DAB Peroxidase Substrate (Vector Laboratories, 1:1000 dilution, 5 min at room temperature). Negative controls included tissue sections incubated with isotype-matched control antibodies and processed with omission of the primary antibody or lectin to assess background and non-specific staining. Sialidase digestion (α-neuraminidase treatment) was performed on selected sections before sTn staining to confirm the specificity of sialylated glycan detection; loss of signal upon treatment validated sialic acid-dependent binding. Positive controls included colorectal tumor tissues previously confirmed to express CD276, Tn, or sTn, as well as *C1GALT1*-knockout CRC cell-derived cells, which are known to exhibit high levels of exposed GalNAc residues for VVA specificity confirmation. Staining was semi-quantitatively evaluated based on both staining intensity (graded 0–3) and extent of positive staining across the tissue Sect. (0–100%). A composite immunoreactivity score was derived by combining intensity and extension to reflect overall expression levels. All scoring was independently assessed by two trained observers and validated by an experienced pathologist to ensure consistency and biological relevance. All slides were imaged using a Motic BA310E microscope and analysed with Motic Images Plus 3.0 software (Motic).

### Double staining immunofluorescence

A subset of FFPE tissue sections previously confirmed to be positive for Tn and CD276 was selected for double immunofluorescence staining to assess the colocalization of both epitopes. Briefly, tissue sections were deparaffinized, rehydrated, and subjected to heat-induced antigen retrieval in 1 mM EDTA buffer (pH 8.0; VWR). Tn antigens were detected by incubation with FITC-labelled VVA at a concentration of 40 μg/mL for 2 h at room temperature. CD276 was detected with an unlabelled rabbit polyclonal anti-CD276 antibody (Thermo Fisher Scientific; 1:250, 1 h at room temperature), followed by an Alexa Fluor 594–conjugated anti-rabbit secondary antibody (30 min at room temperature, in the dark). Controls were consistent with those used for immunohistochemistry, including isotype-matched antibodies and the omission of primary reagents. Additionally, single-staining controls were included to exclude bleed-through or spectral overlap between fluorescence channels and to ensure specific signal attribution for each target. Fluorescence images were acquired in a Leica DMI6000 FFW microscope and analysed with LAS X software (Leica Microsystems).

### Proximity Ligation Assay (PLA)

The PLA was used to detect instances of close spatial proximity (< 30 nm) between CD276 and sTn antigens in colorectal tumor and healthy tissue sections. FFPE tissues previously demonstrating co-localization of CD276 and sTn by immunohistochemistry, including normal colon samples, were selected for analysis. Tissue sections were deparaffinized, rehydrated, and subjected to antigen retrieval in boiling 1 mM EDTA buffer (pH 8.0; VWR) for 20 min. Non-specific binding was blocked by blocking solution (Sigma‒Aldrich) for 1 h at 37 °C. Primary antibodies against CD276 and sTn (anti-TAG-72) were applied and incubated overnight at 4 °C under the same conditions used for immunohistochemistry. PLA signal ligation and rolling circle amplification were performed using the Duolink® In Situ PLA kit (Sigma-Aldrich) according to the manufacturer’s instructions. Sections were then counterstained with DAPI and mounted using an aqueous mounting medium (Bio-Optica). To confirm signal specificity and exclude background noise, multiple controls were included. Tissue sections lacking expression of either CD276 or sTn, as determined by prior immunohistochemistry, were processed in parallel as negative controls. Additional negative controls included sections incubated with only one of the primary antibodies or with both primary antibodies omitted to assess potential non-specific amplification or probe cross-reactivity. Furthermore, selected sections were pre-treated with α-neuraminidase (sialidase) prior to PLA staining to enzymatically remove sialic acids and confirm the sialylation dependence of the sTn signal. Positive controls consisted of colorectal tumor tissues previously validated for co-expression and spatial proximity of CD276 and sTn antigens by immunohistochemistry and immunofluorescence. Fluorescence images were acquired on a Leica DMI6000 FFW microscope and analysed with the LAS X software (Leica Microsystems).

### Statistical analysis

All statistical analyses were performed using GraphPad Prism (v9; Dotmatics), SPSS for MacOS (version 27; IBM), and R software (version 4.2.1; R Foundation for Statistical Computing). For in vitro and ex vivo experiments, including glycogene and *CD276* gene expression (RT‒qPCR), cell invasion and proliferation assays, cytokine production, T cell proliferation, and immune phenotype characterization, statistical comparisons were conducted by one-way ANOVA followed by Tukey’s post hoc multiple comparisons test, which was applied whenever parametric assumptions were met. Data normality was assessed by the Shapiro–Wilk test. When variables deviated from normality, non-parametric tests were used instead, including the Wilcoxon rank-sum test or the Mann-Whitney U test, as appropriate. Chi-square tests were used to evaluate associations between categorical variables such as tumor stage, anatomical location, CD276 status, and CD276–Tn/sTn expression. Differences in CD276 expression and staining extent between metastatic and non-metastatic samples were assessed using a non-parametric t test (Mann–Whitney U test). For TCGA-based transcriptomic analyses, including comparisons between epithelial-like and mesenchymal-like CRC subtypes, non-parametric Mann-Whitney U tests were used to evaluate differences in CD276 and CD276–Tn/sTn expression levels. Spearman’s rank correlation was used to assess associations between gene expression levels, and correlation matrices were visualized using the “corrplot” R package. To explore the prognostic value of CD276 and glycosyltransferase gene expression, univariate and multivariate Cox proportional hazards regression models were performed. Kaplan–Meier survival curves were generated for overall survival (OS) and progression-free survival (PFS), and differences between survival groups were tested using the log-rank test, as implemented in the “survival” and “survminer” R packages. Optimal expression cut-offs for survival stratification were identified using the surv_cutpoint() function. Unless otherwise stated, parametric tests were used only when normality assumptions were satisfied, and non-parametric alternatives were applied otherwise. Statistical significance was considered at *p* < 0.05.

## Results

In this study, we started by investigating the role of immature protein *O*-glycosylation in aggressive CRC. We first analysed glycogene expression patterns in patient samples and then profiled glycan compositions in CRC tissues across differentiation states. Finally, we focused on CD276, a metastasis-associated immune checkpoint glycoprotein, to examine how *O*-glycan remodelling influences tumor cell behavior and immune suppression.

### Transcriptomic shifts in *O*-glycosylation pathways define CRC aggressiveness

**Fig. 1:**
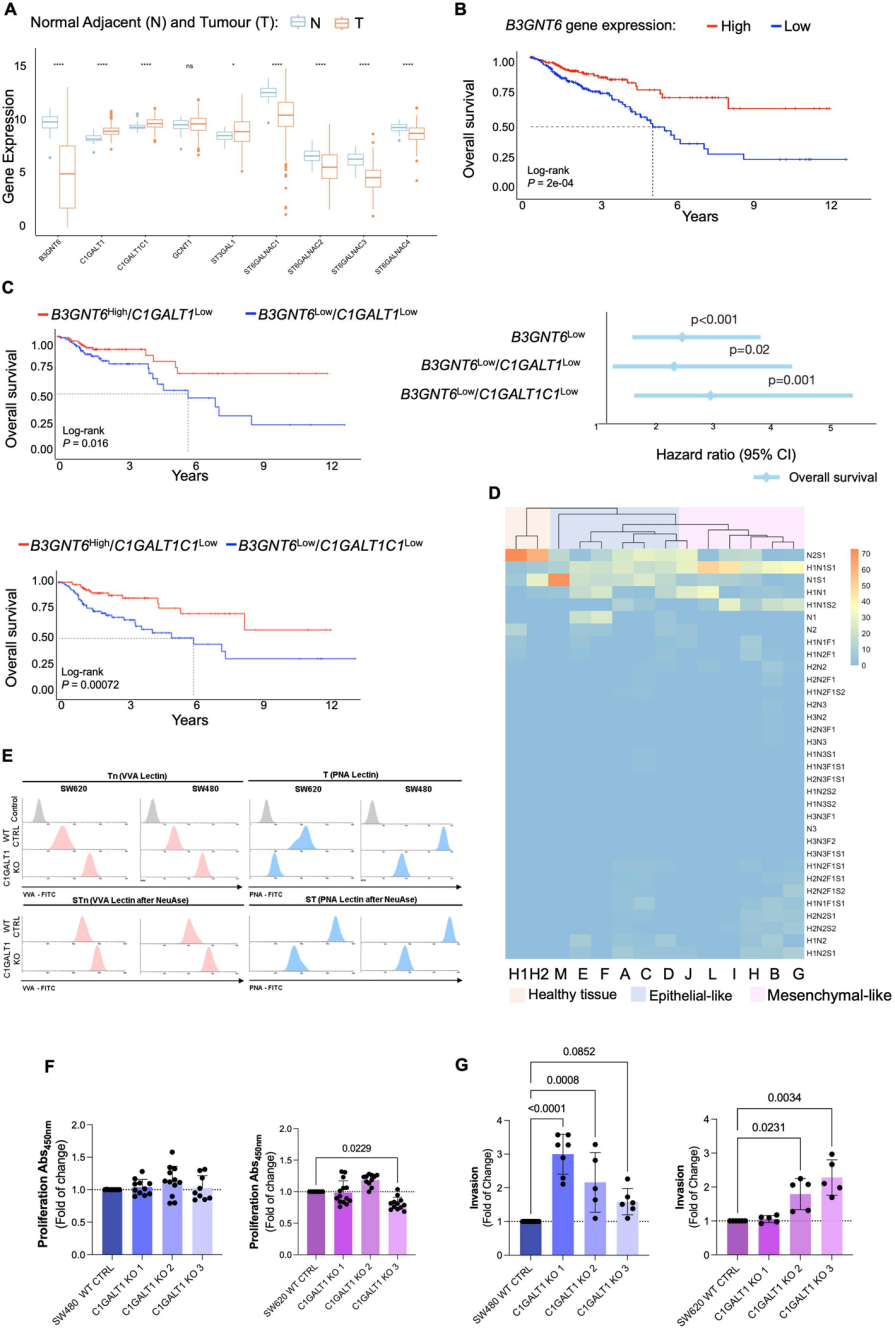
Downregulation of *B3GNT6* and *C1GALT1* promotes immature *O*-glycosylation, defines poor-prognosis CRC subtypes, and enhances tumor cell aggressiveness. **A** Key *O*-glycosylation enzymes are downregulated in colorectal tumors. Transcriptomic analysis of the TCGA-COADREAD dataset comprising 427 samples (376 tumor and 51 normal adjacent tissues) revealed significant downregulation of *B3GNT6* in tumor samples compared to normal tissue (Wilcoxon test, *p* < 0.05), indicating impairment in core 3 *O*-glycan synthesis. ns = not significant; * = *p* < 0.05; **** = *p* < 0.0001. **B** *B3GNT6* expression is associated with patient prognosis. Kaplan–Meier survival analysis showed that lower *B3GNT6* expression is significantly associated with decreased overall survival in CRC patients (log-rank *p* < 0.05). **C** Combined downregulation of *B3GNT6* and *C1GALT1* or *C1GALT1C1* identifies high-risk subgroups. Patients with low *B3GNT6* and either low *C1GALT1* or *C1GALT1C1* expression (associated with an immature glycophenotype) had significantly worse survival (log-rank *p* < 0.05), reinforcing the prognostic relevance of immature *O*-glycosylation profiles. **D** Glycomic profiling distinguishes tumor differentiation states, metastatic potential, and deviation from normal glycosylation. Mass spectrometry-based glycomic analysis revealed that healthy colon mucosa (H1, H2) predominantly expresses sialylated core 3 *O*-glycans. In contrast, epithelial-like tumors are enriched in Tn/sTn antigens, while mesenchymal-like tumors present mono- or di-sialylated core 1 structures. Metastatic tumors exhibit elongated core 2 *O*-glycans irrespective of differentiation state, illustrating the progressive shift from mature to truncated *O*-glycan profiles across CRC progression. Core 3 stuctures: N2, N2S1; Tn: N1; sTn: N1S1; T antigen: H1N1; sialyl-T antigens: H1N1S1, H1N1S2; and extended Core 2-related structures: H1N1F1, H1N2F1, H2N2, H2N2F1, H2N2S1, H2N2S2, H1N2F1S1, H1N2F1S2, H2N2F1S1, H2N2F1S2, N3, H2N3, H3N2, H2N3F1, H3N3, H3N3F1, H3N3F2, H3N3F1S1. **E** *C1GALT1* knockout induces immature *O*-glycans in CRC cells, mimicking tumor-associated glycophenotypes. Flow cytometry analysis of SW480 and SW620 *C1GALT1* KO cells (*n* = 3 independent replicates per condition) revealed reduced PNA binding, both with and without neuraminidase treatment, indicating loss of core 1 and sialylated core 1 glycans, consistent with impaired *O*-glycan elongation. Concurrently, increased VVA binding was observed, revealing accumulation of Tn antigens. After neuraminidase treatment, a further increase in VVA signal confirmed the presence of sTn, demonstrating that many Tn structures are capped by sialic acids. Together, these results validate the induction of a truncated and sialylated *O*-glycophenotype in *C1GALT1*-deficient cells, which closely mimics glycosylation features of colorectal tumors. **F** Immature glycosylation does not affect cell proliferation. *C1GALT1* KO does not significantly change proliferation compared to wild-type controls (n = 15 wells per condition, from 3 independent experiments; one-way ANOVA followed by Tukey’s multiple comparisons test; *p* < 0.05). **G** Immature glycosylation increases tumor cell invasiveness. Matrigel invasion assays increase in invasiveness in both SW480 as well as two clones of SW620 *C1GALT1* KO cells compared to wild type (*n* = 8 wells per condition, from 3 independent experiments). Differences were statistically significant (*p* < 0.05; one-way ANOVA followed by Tukey’s multiple comparisons test)

We began by addressing the expression of key glycogenes encoding glycosyltransferases involved in the initial steps of protein *O*-glycosylation. Analysis of the TCGA dataset, comprising more than 600 CRC cases across all disease stages and normal mucosa samples, revealed significant alterations in glycosylation pathways associated with tumor progression and aggressiveness. Most notably, *B3GNT6*, which initiates core 3 *O*-glycan biosynthesis, was markedly downregulated in CRC (Fig. 1A), suggesting a reduction in core 3-type *O*-glycans in malignant tissues. In parallel, *ST6GALNAC1-4*, which mediate *O*-6 GalNAc sialylation, were consistently decreased in cancer tissues compared to healthy mucosa. In contrast, there was significant upregulation of *C1GALT1* and its chaperone *C1GALT1C1*, which extend glycans beyond the Tn antigen into core 1 structures. *ST3GAL1,* a sialyltransferase that promotes sialyl-T antigen formation, was also upregulated. These findings indicate a biosynthetic shift favouring core 1 over core 3 *O*-glycans in CRC (as highlighted by the schematic *O*-glycosylation pathway representation in Figure S1, Supporting Information). Importantly, low *B3GNT6* expression was strongly associated with poor clinical outcomes, with patients with *B3GNT6*^Low^ tumors showing significantly worse overall survival (*p* = 0.0002; HR = 2.32, *p* < 0.001; Fig. 1B).

To further investigate glycophenotypic patterns, we stratified tumors based on *B3GNT6* expression in combination with low levels of *C1GALT1* or *C1GALT1C1 (*Fig. 1C*)*. Although *C1GALT1* and *C1GALT1C1* are upregulated in CRC (Fig. 1A), their reduced expression, when coupled with low *B3GNT6*, may limit core 1 and core 3 synthesis, leading to immature, cancer-associated glycosylation. Consistent with this, tumors displaying a *B3GNT6*Low/*C1GALT1*Low or *B3GNT6*Low/*C1GALT1C1*Low profile showed the poorest prognosis (*B3GNT6*^Low^/*C1GALT1*^Low^: HR = 2.18, *p* < 0.02; *B3GNT6*^Low^/*C1GALT1C1*^Low^: HR = 2.79, *p* < 0.001; Fig. 1C). Together, these findings suggest that CRC progression is linked to simple glycophenotypes, marked by the accumulation of Tn and, potentially sTn antigens, which align with more aggressive disease and poorer clinical outcomes.

### Differentiation-linked glycome profiles reveal simple glycophenotypes in advanced CRC

To explore whether these transcriptional alterations translate into distinct glycan phenotypes, we next profiled the tissue glycome in healthy mucosa and advanced CRC (stage ≥ 3). Our analysis focused how tumor differentiation shapes *O*-glycosylation patterns.

The tumors were further characterized based on their differentiation status, a feature often linked to consensus molecular subtypes (CMSs) [35]. Accordingly, we categorized the tumors into two groups: i) differentiated epithelial tumors, characterized by high CDX2 and low FRMD6 and HTR2B expression levels (Figure S2a), which typically correspond to the CMS2 and CMS3 subtypes, and ii) undifferentiated mesenchymal tumors, characterized by low CDX2 and high FRMD6 and HTR2B levels, which are generally associated with CMS1 or CMS4 [36].

No differences in survival were observed between these groups (Figure S2a), likely reflecting the uniformly advanced stage of disease in the cohort. In healthy mucosa, the glycome was primarily composed of core 3 structures (N2, N2S1; Fig. 1D, Figure S3, Table S1 and Table S2), predominantly decorated with *O-6-GalNAc-*linked sialic acids (Figure S3). The sTn antigen (N1S1) was also present at relatively low levels, which is consistent with previous reports [37]. These features align with the increased expression of the *B3GNT6* and *ST6GALNAC* sialyltransferase genes in healthy colon tissue (Fig. 1A). In contrast, tumor samples presented significantly lower levels of core 3 glycans and were predominantly enriched in sialyl-T antigens (H1N1S1) and di-sialyl-T antigens (H1N1S2; Fig. 1D). Most tumors expressed Tn (N1) and/or sTn (N1S1), whereas only a small subset, mainly mesenchymal-like tumors, exhibited extended core 2 *O*-glycans (H2N2, H2N2F1, H1N2F1S2, H1N2S1, H2N2S2; Fig. 1D). Nevertheless, a striking difference emerged between epithelial-like and mesenchymal-like tumors. Epithelial-like tumors exhibited higher levels of core 3 and sTn and de novo expression of Tn antigens, which was consistent with a shift towards simpler glycophenotypes (Fig. 1D, Table S2). In contrast, mesenchymal-like tumors were enriched in mono- and di-sialylated T antigens, with low relative expression of other glycan structures. Notably, all tumors with distant metastases, regardless of their differentiation status, exhibited multiple sialylated core 2-related structures (H1N2S1, H2N2S2, H2N2S1, H2N2F1S2; Fig. 1D). No additional associations were observed between tumor stage, grade, patient survival, or glycome composition (data not shown). Complementary immunohistochemistry confirmed the presence of Tn and/or sTn antigens in 90% of primary tumors (Table S3), in agreement with MS analysis. Interestingly, while the relative abundance of these glycans varied between epithelial- and mesenchymal-like tumors (Fig. 1D), immunohistochemistry analysis revealed that the overall expression levels of Tn and sTn remained consistent across differentiation states (data not shown). Notably, the expression of sTn showed an association with the T stage (Table S3).

In summary, these findings highlight distinct glycome profiles between tumor differentiation states in aggressive CRC. Immature glycosylation patterns were prevalent across advanced tumors, including lymph node and distant metastases. Epithelial-like lesions displayed an intermediate glycophenotype, situated between healthy colonic mucosa and mesenchymal-like tumors. This profile was marked by a partial loss of core 3 structures, reduced glycan complexity, and increased expression of Tn and sTn antigens. In contrast, mesenchymal tumors were more enriched in sialylated core 1 and extended core 2 glycans. Despite these subtype-specific differences, simple glycophenotypes, particularly Tn and sTn, were widely present across tumor types, with their relative abundance shaped by differentiation status. These findings align with transcriptomics data, confirming that downregulation of core 3–associated enzymes in CRC (Fig. 1A). They further support the presence of simple glycophenotypes driven by reduced *C1GALT1* and *C1GALT1C1* expressions, which vary with tumor differentiation and associate with disease progression.

### Simple glycophenotypes enhance CRC cell invasiveness

To explore the functional role of simple glycophenotypes, we first profiled the *O*-glycome of a panel of CRC cell lines representative of epithelial-like (HCA7, SW480) and mesenchymal-like (SW620, HCT116, RKO, LS174T) phenotypes (Figure S4 and Table S4). These profiles closely mirrored those of human tumors, with epithelial-like cells predominantly expressing core 3 glycans and sialylated T antigens, while mesenchymal-like lines were mainly enriched in core 1 structures. Notably, sTn antigens were absent in epithelial-like cells, suggesting that their expression in tumors may be influenced by the tumor microenvironment.

To experimentally induce simple glycophenotypes, we knocked out *C1GALT1* using CRISPR-Cas9 in both SW480 (epithelial-like, derived from a primary tumor) and SW620 (mesenchymal-like, from a lymph node metastasis of the same patient) cell lines (Figure S5). This isogenic pair derives from the same patient, reducing inter-patient genomic variability and allowing comparison between matched primary and metastatic stages of CRC. Despite their differing baseline glycomes, *C1GALT1* knockout completely suppressed core 1 structures in both cell lines and led to robust overexpression of Tn and sTn antigens (Fig. 1E, Figure S6). Additionally, both cell lines presented a marked decrease core 3 glycans. These changes were accompanied by reduced expression of *B3GNT6* (Figure S5C), suggesting a degree of co-regulation between core 1 and core 3 glycosyltransferases, mimicking the *C1GALT1*^Low^/*B3GNT6*^Low^ phenotype associated with poor prognosis in patient samples (Fig. 1B-C). Both SW480 and SW620 C1GALT1 knockout cells showed no change in proliferation compared to controls (Fig. 1F) but exhibited increased matrigel invasion in vitro (Fig. 1G). Collectively, these findings demonstrate that the acquisition of simple glycophenotypes, marked by the loss of core 1 and core 3 glycans and accumulation of Tn/sTn antigens, enhances the invasive potential of CRC cells independently of proliferation. This glycome remodelling mirrors the poor-prognosis transcriptional signatures identified in patient tumors and highlights a functional role for immature *O*-glycans in promoting CRC aggressiveness.

### CD276 carries immature *O*-glycans in CRC and associates with poor prognosis

**Fig. 2:**
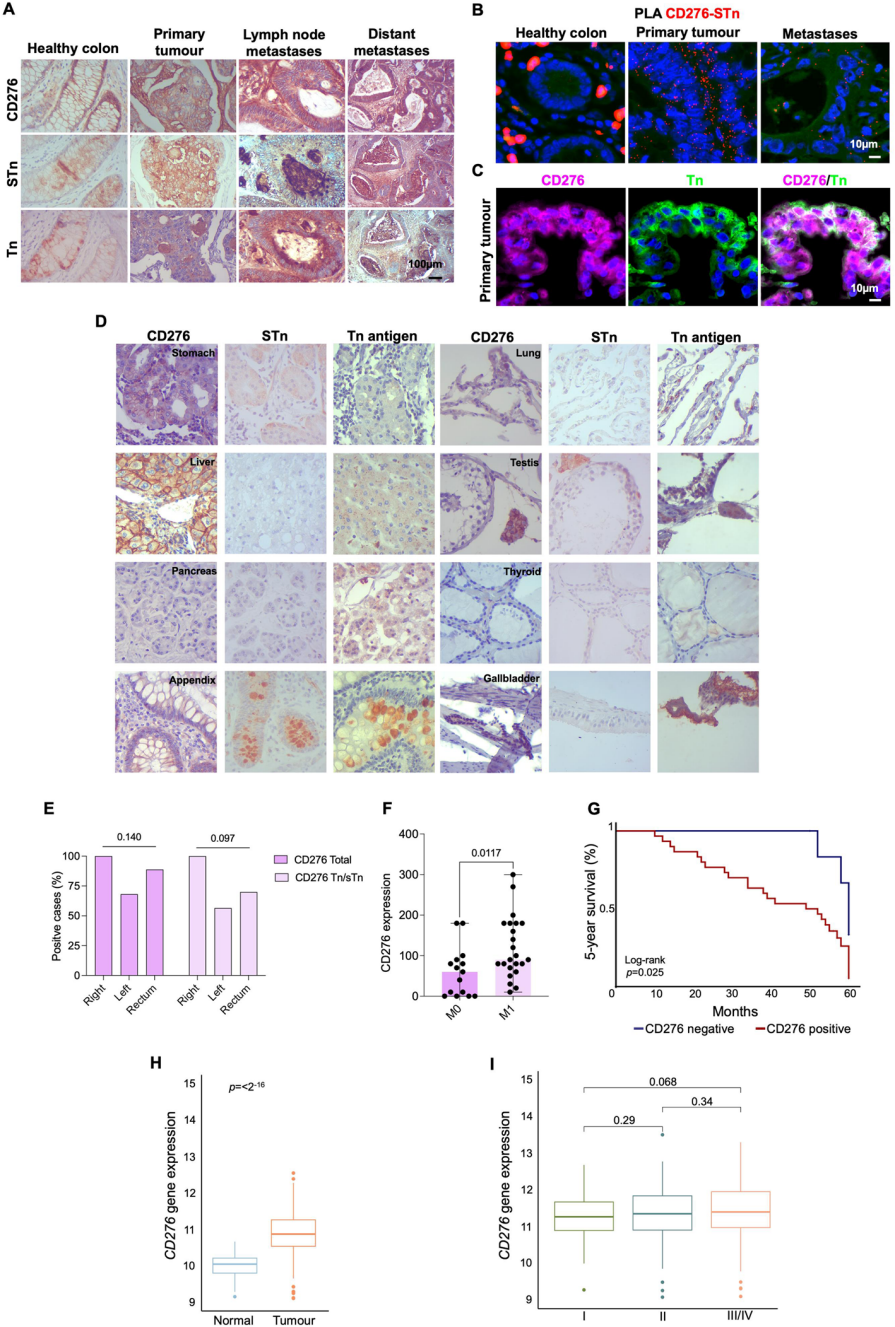
CD276 is a marker of poor prognosis in colorectal cancer and displays cancer-specific aberrant *O*-glycosylation. **A** CD276 colocalizes with Tn and sTn antigens in CRC and metastases. Immunohistochemistry reveals diffuse CD276 expression throughout tumor regions, both in the cytoplasm and at the cell membrane, overlapping with Tn and sTn antigen distribution. Unlike sTn, CD276 is also detected in the extracellular matrix surrounding tumor areas. Tn expression appears in scattered niches without a consistent pattern. In contrast, the healthy colon shows only weak cytoplasmic staining for CD276, Tn, and sTn, with no evidence of colocalization. **B** In situ proximity ligation assay (PLA) confirms CD276-sTn glycoproteoforms in CRC and metastases. PLA reveals strong spatial proximity (< 30 nm; red puncta) between CD276 and sTn in tumor cells from both primary tumors and metastases, but not in healthy colon, highlighting the cancer-specific nature of this aberrant glycoform. **C** Double immunofluorescence reveals CD276-Tn glycoproteoforms in CRC and metastases. In CD276-enriched tumors, CD276 (magenta) and Tn (green) co-localize at both the membrane and cytoplasm of the same cells, confirming CD276 as a direct carrier of immature O-glycans. **D** Aberrant CD276 glycoforms are cancer-specific. Immunohistochemistry analysis of healthy tissues reveals limited CD276 expression, primarily cytoplasmic in select epithelial cells (e.g. enterocytes, hepatocytes, Leydig cells), and no co-localization with Tn or sTn antigens except occasional overlap in Leydig cells. In contrast to tumors, sTn and Tn were either absent or restricted to secretory or immune compartments, supporting the tumor-specific nature of CD276-Tn/sTn glycoforms. **E** CD276 and its aberrantly glycosylated forms are enriched in right-sided colorectal tumors CD276 and CD276-Tn/sTn glycoform prevalence across tumor locations revealed anatomical distinctions in both expression and glycosylation. In the right colon, 100% of tumors were CD276-positive and all exhibited aberrant glycosylation with Tn and/or sTn antigens. These expressions were trendily lower for the left side colon and rectum. (Chi-square) **F** CD276 expression is elevated in metastatic CRC. IHC quantification shows significantly higher CD276 scores in M1 (metastatic) versus M0 (non-metastatic) tumors (*p* = 0.0117, Mann–Whitney test). **F** CD276 expression is associated with poor prognosis. Kaplan–Meier survival analysis reveals significantly worse 5-year survival in patients with CD276-positive tumors compared to CD276-negative cases (*p* = 0.025, log-rank test). **H** *CD276* is significantly upregulated in CRC compared to matched normal tissue. TCGA analysis of tumor samples and adjacent normal tissues shows robust overexpression of CD276 in tumors (*p* < 0.0001, Wilcoxon test), confirming its cancer-associated expression profile. **I** *CD276* expression increases with tumor stage. TCGA data supports a progressive increase of CD276 expression from stage I to stage III/IV (*p* = 0.068, one-way ANOVA)

We next investigated whether CD276 could act as a carrier of immature *O*-glycans in CRC. Using immunohistochemistry, we confirmed CD276 expression in approximately 80% of primary advanced CRC tumors, as well as in corresponding lymph node and distant metastases. Notably, CD276 co-localized with areas of high Tn and/or sTn expression in all CD276-positive tumors (Fig. 2A). In healthy colon tissue, although some apparent co-localization was observed, CD276 was primarily expressed in enterocytes, whereas Tn and sTn antigens were restricted to goblet cells and were much less abundant than in tumors (Fig. 2A). To further assess whether CD276 directly carried these immature glycans, PLA and immunofluorescence analyses were performed. Both approaches provided positive signals in CRC samples, supporting the notion that CD276 is a major carrier of Tn and sTn antigens in cancer (Fig. 2B-C). In contrast, no evidence of abnormal CD276 glycosylation was found in healthy colon tissue or in other normal tissues, including stomach, liver, pancreas, appendix, gallbladder, lung, testis and thyroid (Fig. 2D), suggesting a cancer-specific nature.

Clinically, CD276-expressing immature glycosylation was more frequently observed in right-sided colon tumors than in left-sided and rectal lesions (Fig. 2E), a subgroup associated with more aggressive clinical behavior. It was also enriched in metastatic tumors (*p* = 0.0117; Fig. 2F). Furthermore, CD276-positive tumors presented significantly worse prognosis compared to CD276-negative lesions (*p* = 0.0025; Fig. 2G). Interestingly, no differences were observed in the frequency of CD276 expression or in the prevalence of its abnormally glycosylated forms between epithelial-like and mesenchymal-like tumors (data not shown).

Having established CD276 as a potential cancer-specific carrier of immature *O*-glycans, we next explored its gene expression profile. TCGA analysis revealed that CD276 was significantly upregulated in CRC compared to adjacent normal mucosa (Fig. 2H). Moreover, CD276 expression showed a trend toward higher levels in more advanced tumor stages (*p* = 0.07 for stage III/IV; F ig. 2I), reinforcing the link between CD276 expression and poor prognosis.

### CD276 exhibits distinct glycocodes across different states of CRC differentiation

**Fig. 3:**
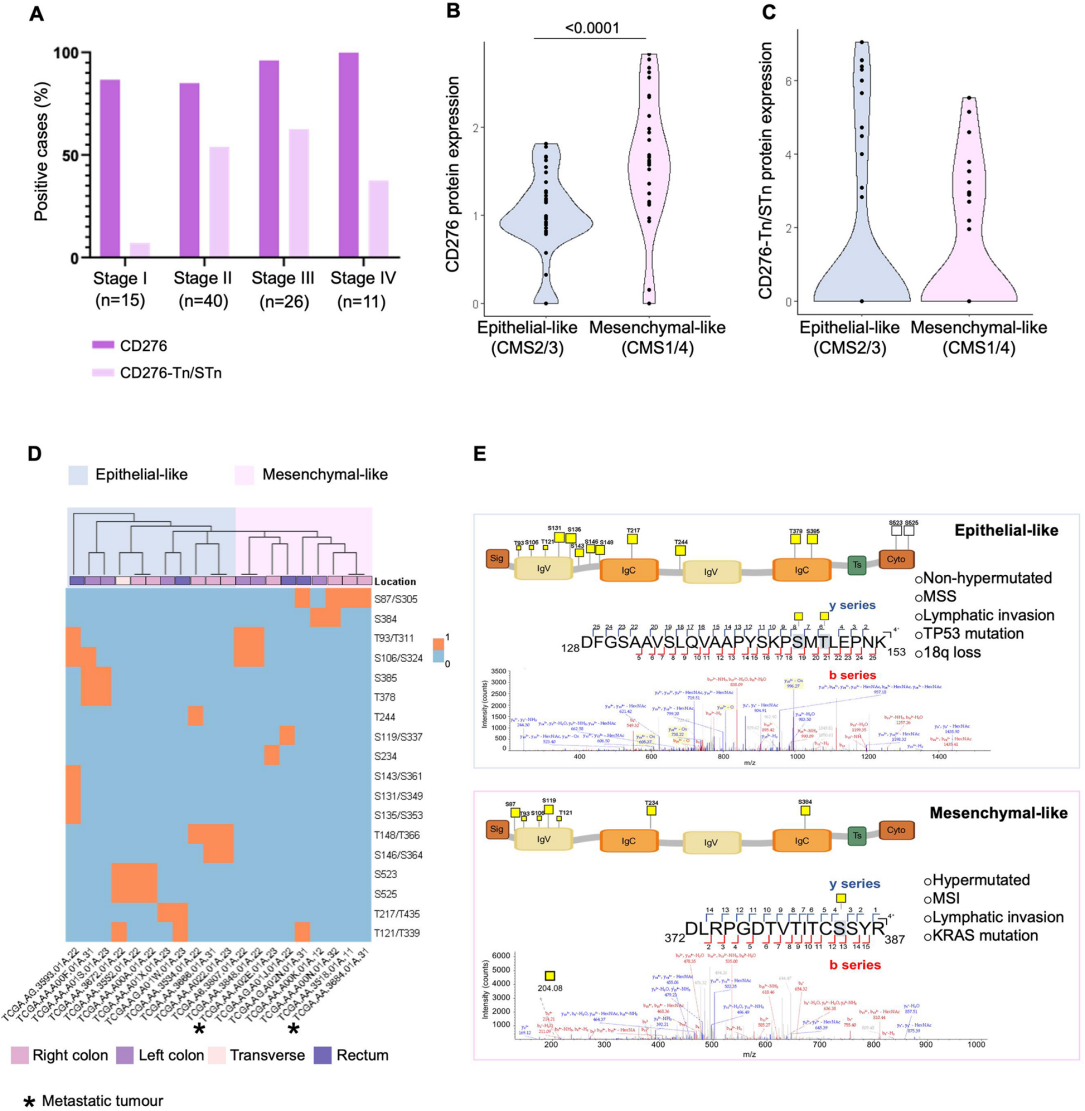
CD276 is widely expressed across CRC stages and displays distinct glycosylation patterns according to tumor differentiation. **A** CD276 is broadly expressed across colorectal cancer stages, while immature CD276 glycoforms (Tn/sTn-modified) are enriched in advanced tumors. The plot concerning TCGA-based proteomics data shows the percentage of positive cases for total CD276 and CD276 glycosylated with Tn/sTn antigens across CRC stages I–IV. Aberrant CD276 glycoforms were significantly more frequent in stages II–IV compared to stage I (Fisher’s exact test, *p* = 0.0026). **B** CD276 expression is associated with tumor differentiation state. Mesenchymal-like tumors (CMS1/4) exhibited significantly higher CD276 protein expression than epithelial-like tumors (CMS2/3) (*p* < 0.0001, Wilcoxon test). **C** Glycosylated CD276 is not significantly associated with tumor subtype. No difference in CD276-Tn/sTn glycoform expression was observed between epithelial-like and mesenchymal-like tumors. **D** CD276 glycosylation patterns differ between epithelial-like and mesenchymal-like colorectal tumors. Heatmap showing the distribution of inferred O-glycosylation sites across tumor samples, stratified by transcriptomic subtype (CMS2/3: epithelial-like; CMS1/4: mesenchymal-like). Each row represents an amino acid position, and each column a tumor; coloured squares indicate detected glycosylation. Epithelial-like tumors display broader glycosylation coverage, with sites clustered within the IgV and IgC domains, whereas mesenchymal-like tumors exhibit sparser and more membrane-proximal modifications. **E** Representative glycopeptides identified by CID MS/MS highlight subtype-specific O-glycosylation within the extracellular domain of CD276. In epithelial-like tumors (top), glycosylation was denser and predominantly located between the IgV and IgC domains. In contrast, mesenchymal-like tumors (bottom) showed sparser and more membrane-proximal glycosylation. Annotated MS/MS spectra display HexNAc-retaining b- and y-ions supporting glycopeptide identification despite CID-related limitations in site resolution. Schematic domain maps indicate glycosite positions, and subtype-associated molecular features are also summarized

To further dissect the glycocode of CD276, we interrogated a well-characterized proteomics dataset from the PRIDE repository. This comprised 95 CRC patients, stratified by tumor stage, location, and differentiation status according to the CMSs. Protein identifications were derived from individual search result files for each sample (Supporting Data File 1). Although the dataset was not specifically optimized for glycoprotein detection, it provided valuable insights into CD276 glycosylation patterns across CRC subtypes. Over 80% of these tumors were positive for CD276, with widespread expression across all stages of the disease (Fig. 3A). Most tumors in stages III and IV exhibited CD276 positivity (Fig. 3A). Notably, the proportion of abnormally glycosylated CD276 (Tn/sTn glycoforms) was significantly higher in stage II-IV tumors compared to stage I (40-60% vs. 5%; Fig. 3A). When stratified by tumor differentiation states, mesenchymal-like tumors (CMS1/4) exhibited significantly higher CD276 protein expression compared to epithelial-like tumors (CMS2/3) (*p* < 0.0001; Fig. 3B). However, no significant differences were observed in the expression of abnormally glycosylated CD276 forms between epithelial- and mesenchymal-like subtypes (Fig. 3C, Figure S7 and Supporting Data File 2). Furthermore, tandem mass spectrometry revealed distinct CD276 glycosylation patterns associated with tumor differentiation, independent of anatomical location or stage (Fig. 3D). CID-MS/MS analysis also enabled high-confidence localization of several immature *O*-glycans on CD276 (Fig. 3D-E, Supplementary Figure S7; Data File 2). In the IgV-like domain, O-glycosylation was consistently assigned to Ser87, Ser106, Ser119, Ser131, Ser135, Ser143 and Ser146. However, each of these residues has a corresponding position in the second repeat of this domain in Ser305, Ser324, Ser337, Ser349, Ser353, Ser361 and Ser364, generating sequence-identical peptides that do not allow unequivocal localization. In contrast, within the IgC-like domain, O-glycosylation at Thr148/Thr366 was unambiguously localized. Further confidently assigned modifications included Thr378, located immediately upstream of the C-terminal hinge, Ser385 within the proximal stalk region, and Ser523 and Ser525 near the acidic C-terminal segment. Several other Ser/Thr-rich motifs generated reproducible HexNAc-containing spectra but lacked the ion coverage necessary for fully confident residue-level discrimination. These included Thr93/Thr311, Thr121/Thr339, Thr217/Thr435, Ser234, Thr244 and Ser384. Although CID cannot distinguish individual *O*-glycosylated residues, the consistency of HexNAc-retaining fragments across multiple glycopeptide spectra strongly supports their classification as high-confidence *O*-glycosylated regions. Definitive localization will require future validation by electron-based fragmentation (ETD/EThcD). Despite these limitations, MS data reinforce the presence of immature *O*-glycosylation on CD276, as multiple HexNAc-containing glycopeptides were consistently detected across multiple samples (Fig. 3D–E, Supplementary Figure S7 and Data File 2). These observations are also in agreement with our immunoassay and align with subtype-specific glycosylation patterns. Furthermore, the overall distribution of glycopeptide signals across the CD276 sequence allowed us to infer broader patterns of glycosylation occupancy. Notably, in epithelial-like tumors, glycosylation appeared more abundant and clustered along the immunoglobulin variable (IgV) and constant (IgC) domains of CD276 (Fig. 3E). In contrast, mesenchymal-like tumors exhibited sparser and more membrane-proximal glycosylation. Interestingly, *O*-glycosylation site prediction using NetOGlyc identified only three putative sites, including T244, which was also supported by our analysis. However, MS-based profiling revealed a substantially broader and phenotype-dependent glycosylation landscape, underscoring the limitations of prediction tools and the added value of experimental mapping (Fig. 3E). Taken together, these results indicate that CD276 glycosylation is linked primarily to tumor differentiation rather than to anatomical location or metastatic status. It also provides a foundation for understanding its functional role in cancer and for guiding therapeutic targeting strategies.

### CD276 overexpression is driven by immature glycosylation

**Fig. 4:**
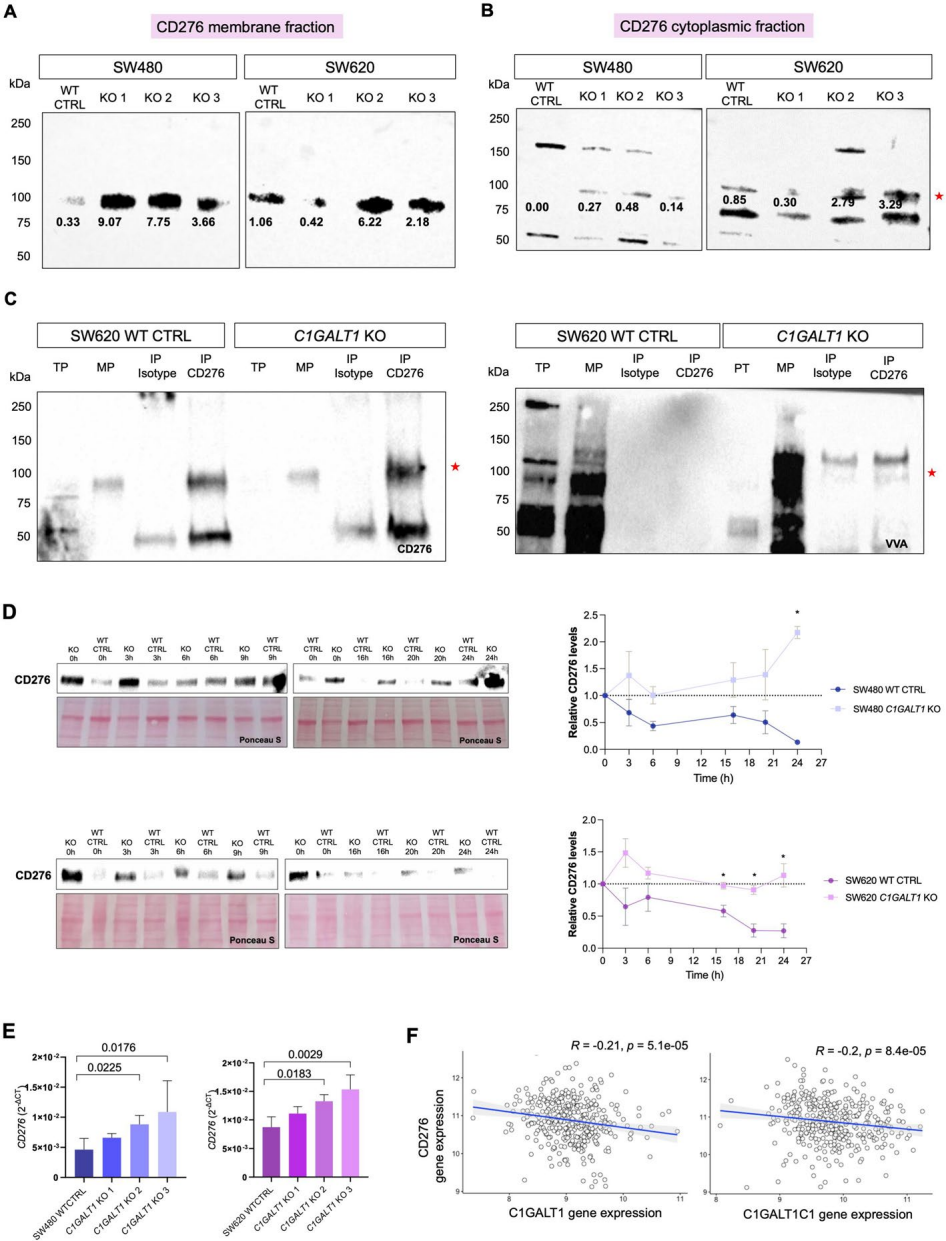
Loss of *C1GALT1* expression increases CD276 expression and results in the accumulation of immature Tn-modified glycoforms. **A**-**B** Loss of *C1GALT1* leads to an increase in CD276 protein levels in CRC cells. Western blot analysis was performed on total protein lysates (T) and plasma membrane fractions (PM) from SW480 and SW620 wild-type (WT) and *C1GALT1 *knockout (KO) cells using antibodies against either the extracellular (**A**) or cytoplasmic (**B**) domains of CD276. Bands were normalized to Ponceau S and are highlighted in bold in the blots. KO cells showed increased CD276 protein expression compared to WT. A prominent 100 kDa band (red asterisk), compatible a CD276 glycoproteoform, was observed in *C1GALT1 *KO cells. **C** Immunoprecipitation of CD276 confirms increased protein levels and enrichment of a 100 kDa glycoform in *C1GALT1* KO cells. CD276 was immunoprecipitated from plasma membrane fractions of SW620 WT and KO cells. Western blotting detected a clear ~ 100 kDa band in *C1GALT1 *KO samples, which was absent in isotype controls and enhanced relative to WT. This result confirms successful IP and increased expression of CD276 glycoproteoforms in KO cells. VVA lectin blotting was also performed to assess Tn antigen on CD276, confirming the presence of immature glycoforms in CD276 IPs. Ponceau S staining was used to verify equal protein loading. **D** Cycloheximide treatment reveals enhanced CD276 protein stability in *C1GALT1 *knockout cells. SW480 and SW620 wild-type and *C1GALT1 *KOs were treated with cycloheximide to block protein synthesis, and CD276 levels were analyzed by immunoblotting overtime. Quantification shows significantly increased CD276 stability in SW620 KO cells compared to WT (*p* < 0.05), and a similar non-significant trend in SW480 cells, indicating that loss of *O*-glycan elongation stabilizes CD276 post-translationally (Two-way ANOVA with uncorrected Fisher’s LSD post-hoc test; *p < 0.05). **E** Loss of *C1GALT1 *leads to transcriptional upregulation of CD276. In both cell lines, CD276 gene expression was significantly elevated in *C1GALT1* KO clones compared to WT controls. Each bar represents an independent biological *C1GALT1 *KO clone. Values correspond to the mean of technical triplicates. Statistical significance was determined using one-way ANOVA followed by Tukey’s multiple comparison test. **F** Reduced expression of core 1 *O*-glycosylation enzymes is associated with increased CD276 expression in CRC. TCGA transcriptomic analysis revealed that CD276 gene expression inversely correlates with that of C1GALT1 (R = –0.21, *p* = 5.1 × 10⁻5) and C1GALT1C1 (R = −0.20, *p* = 8.4 × 10⁻5). These results support a negative association between core 1 *O*-glycan biosynthesis and CD276 expression in CRC, consistent with increased CD276 levels and immature glycosylation under reduced *C1GALT1* expression

We next investigated CD276 glycoproteoforms in both wild-type and *C1GALT1* knockout CRC cell models. Immunoblotting with an antibody directed against the membrane portion of CD276 revealed an increase in the 100 kDa band following *C1GALT1* knockout in both SW480 and SW620 cells (Fig. 4A). A similar pattern was observed using an antibody targeting the cytoplasmic domain (Fig. 4B). The 100 kDa band is consistent with the full-length CD276 isoform containing two IgV–IgC domain pairs, with the increased apparent molecular weight reflecting extensive glycosylation. These findings indicate that *C1GALT1* knockouts consistently lead to the accumulation of a densely glycosylated CD276 form, independent of the antibody epitope targeted. Additional lower molecular weight bands were detected with the cytoplasmic domain-targeting antibody (Fig. 4B), potentially representing alternative isoforms; however, these bands did not show consistent glycosylation patterns. To further validate the presence of immature *O*-glycans, CD276 was immunoprecipitated from wild-type and *C1GALT1* knockout cells. Isotype control immunoprecipitations showed no detectable bands, confirming the specificity of CD276 pulldown. In a validation experiment using SW620 cells, the predominant 100 kDa CD276 band was detected at higher intensity after immunoprecipitation. It also showed strong VVA lectin reactivity, confirming Tn accumulation on CD276 following disruption of glycan extension (Fig. 4C and Figure S8). To evaluate whether *C1GALT1* knockout affects CD276 stability, cells were then treated with cycloheximide to block protein synthesis. CD276 levels were then tracked over time. In wild-type cells, CD276 levels declined as expected (Fig. 4D). In contrast, knockout cells showed delayed degradation, indicating increased protein stability. This effect was statistically significant in SW620 cells (*p* < 0.05) and showed a similar, though non-significant, trend in SW480 cells (Fig. 4D). These results suggest that loss of core 1 O-glycosylation impairs CD276 turnover, enhancing its post-translational stability.

Finally, we examined CD276 transcript levels, which were significantly increased in *C1GALT1* knockout cells compared to wild-type controls in both cell lines (Fig. 4E). Analysis of patient TCGA datasets further revealed a significant inverse correlation between *CD276* expression and both *C1GALT1* and *C1GALT1C1* expressions (Fig. 4F). This supports the transcriptional co-regulation mechanisms suggested by our glycoengineered cell lines, linking immature *O*-glycosylation to CD276 upregulation. Similar observations were made for *GCNT1*, involved in core 2 formation, reinforcing a link with immature glycosylation. In addition, CD276 associated with increased expression of *ST3GAL1*, *ST6GALNAC2*, and *ST6GALNAC3*, suggesting a shift toward enhanced sialylation of immature *O*-glycans, particularly Tn and core 1 structures (Figure S8). These observations point to broader, previously unrecognized regulatory networks linking *O*-glycosylation remodelling and CD276 expression in CRC, warranting further investigation.

Collectively, these findings demonstrate that immature *O*-glycosylation driven by *C1GALT1* loss promotes CD276 overexpression through combined effects on protein stability and gene transcription, highlighting a mechanistic link between cancer glycome remodelling and CD276 regulation in CRC.

### CD276 immature glycosylation drives cancer cell aggressiveness and oncogenic signalling

**Fig. 5:**
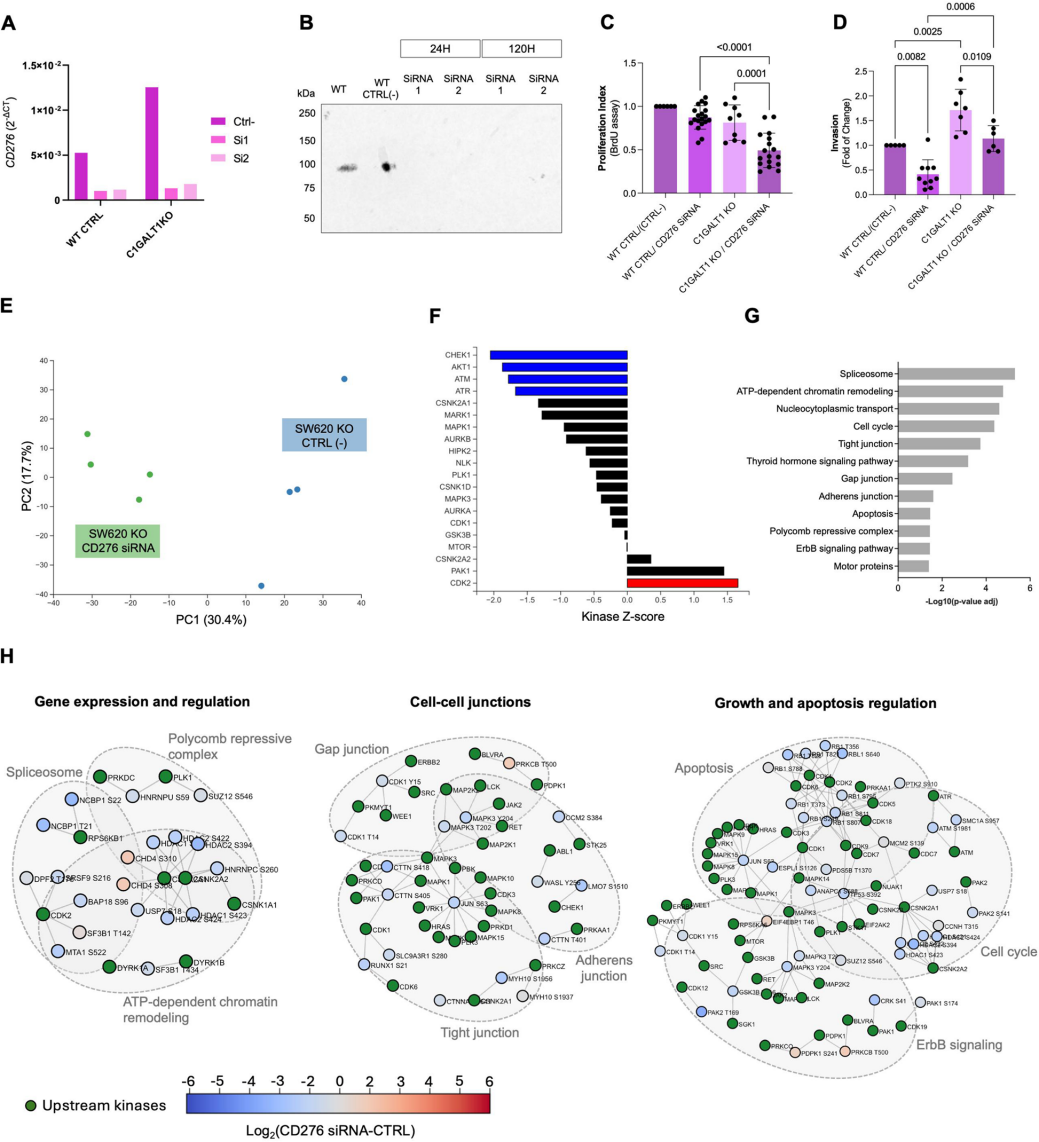
**A-B** *CD276* silencing using siRNA effectively reduces gene and protein expression in both wild-type and *C1GALT1* KO CRC cells. qPCR shows ~ 85% knockdown of *CD276* transcripts with two independent siRNAs (Si1 and Si2) in both cell models compared to non-targeting control (Ctrl-). Western blot analysis confirms complete abrogation of CD276 protein expression up to 5 days post-transfection. C. Aberrantly glycosylated CD276 supports CRC cell proliferation in SW620 cells. In SW620 cells, *CD276* silencing significantly reduced proliferation in *C1GALT1* KO cells in relation to controls (*p* = 0.0001; one-way ANOVA followed byTukey’s multiple comparisons test). Data represents the mean fold change relative to control from three independent experiments. **D** Aberrantly glycosylated CD276 promotes CRC cell invasion in SW620 cells. CD276 silencing decreased invasion in both wild-type and *C1GALT1*-deficient cells. Notably, in *C1GALT1* KO cells CD276 knockdown reversed the glycosylation-induced increase in invasion, confirming a glycan-dependent pro-invasive role. Data represents fold change relative to control and is derived from three independent experiments (one-way ANOVA followed by Tukey’s multiple comparisons test). **E-H** Aberrantly glycosylated CD276 sustains phospho-signaling programs that promote proliferation, cytoskeletal remodelling in SW620 cells. Phosphoproteomic profiling was performed on glycoengineered SW620 CRC cells expressing Tn-modified CD276, with or without *CD276* knockdown. CD276 glycosylation was previously associated with increased proliferation and invasion; here, mass spectrometry and Phosphomatics-based analysis reveal that these phenotypes are underpinned by distinct phospho-regulatory networks. **E** Immature glycosylated CD276 supports a distinct phospho-signaling landscape. PCA of phosphopeptide intensities revealed a clear separation between *CD276*-silenced and control cells, indicating widespread remodelling. **F** Abnormal CD276 glycosylation promotes kinase signalling programs linked to proliferation and cytoskeletal organization. Kinase-substrate enrichment analysis (KSEA) revealed reduced activity of kinases such as CHEK1, AKT1, ATM and ATR upon *CD276* knockdown, indicating that these proliferative and motility-associated kinases are supported by immature CD276 glycosylation. In contrast, kinases involved in checkpoint control and stress responses, including CDK2, were activated following *CD276* silencing, consistent with induction of growth restraint and DNA damage surveillance mechanisms. **G** Abnormal CD276 glycosylation maintains signaling across pathways involved in chromatin remodeling, cytoskeletal integrity, and cell survival. Pathway enrichment analysis of differentially phosphorylated proteins revealed that *CD276* knockdown suppresses phospho-regulated processes such as spliceosome and chromatin remodeling complexes (e.g., NuRD), tight junction organization, nucleocytoplasmic transport, and apoptotic resistance. These programs collectively support enhanced plasticity, proliferation, and invasive behavior observed in SW620 *C1GALT1* KO cells. **H** Abnormal CD276 glycosylation maintains phosphorylation of regulatory hubs that drive proliferation, invasion, chromatin remodeling. Phosphomatics network analysis identified distinct phospho-signaling modules dependent on glycosylated CD276, which were disrupted upon its silencing. These include: i) Chromatin regulators (HDAC1/2, CHD4, CSNK2A2), promoting transcriptional reprogramming and epigenetic adaptability; ii) Cytoskeletal and junction-associated proteins (CTTN, MAPKs), supporting cell motility and invasiveness; iii) Growth and survival kinases (RB1, MAPK3), reflecting pathways that enable tumor persistence and proliferation. Together, these data demonstrate that aberrant CD276 *O*-glycosylation drives SW620 pro-oncogenic phosphorylation programs that coordinate enhanced proliferation and invasion

We used SW620 cells, a metastatic model with mesenchymal traits, to explore how aberrant CD276 glycosylation drives tumor aggressiveness in the context of advanced disease. *CD276* was transiently knocked down using two siRNAs (si1 and si2) in SW480 and SW620 wild-type cells, as well as in their corresponding *C1GALT1* knockout models (Fig. 5A). Effects on proliferation and invasion were then assessed (Fig. 5B-C). Efficient knockdown was confirmed by a strong reduction in *CD276* mRNA levels and near-complete loss of the CD276 protein within 24 h, which was sustained for at least 5 days (Fig. 5A and B). CD276 knockdown significantly reduced proliferation in *C1GALT1-*deficient cells but had no effect in wild-type cells, indicating that immature O-glycosylation enhances the pro-proliferative function of CD276. Silencing CD276 also decreased invasion across both backgrounds, confirming a baseline pro-invasive role. Notably, in *C1GALT1*-deficient cells, CD276 knockdown fully reversed the glycosylation-induced increase in invasion, demonstrating that aberrant glycosylation specifically drives CD276-dependent invasiveness.

To dissect the mechanistic events supporting these findings, we also performed phosphoproteomic profiling of SW620 *C1GALT1* knockout cells with or without CD276 knockdown. Principal component analysis (PCA) revealed distinct phospho-signalling profiles between CD276-silenced and control glycoengineered cells (Fig. 5E). Kinase–substrate enrichment analysis highlighted significantly reduced activity (negative z scores) of ATR, ATM, AKT1, and CHEK1, which are key regulators of DNA damage response, checkpoint control, and pro-survival signalling (Fig. 5F) [38–40]. Inhibition of ATR, ATM, and CHEK1 may compromise DNA repair and genotoxic stress responses, whereas reduced AKT1 activity is likely to impair cytoskeletal dynamics and cell adhesion [41]. These results suggest that CD276 sustains kinase-driven programs that support migration, survival, and immune evasion. Interestingly, CDK2 activity showed a modest increase after CD276 knockdown, potentially reflecting compensatory activation of cell cycle progression. Pathway enrichment analysis confirmed the role of CD276 in regulating phosphorylation-dependent networks. These included spliceosome assembly, chromatin remodelling, cell cycle progression, and cytoskeletal organization (Fig. 5G). Collectively, CD276 knockdown led to a coordinated downregulation of kinase activity and downstream signalling modules governing DNA repair, adhesion, and transcriptional regulation, which may reduce tumor cell adaptability and resilience. These molecular alterations were evident in substrate-centred network maps (Fig. 5H) and align with the phenotypic consequences observed in CD276 loss-of-function models (Fig. 5C and D), including reduced proliferation and impaired migration. It further suggests weakened immune evasion, which warrants deeper investigation. Network-based mapping of phosphosite changes further reinforced these findings, revealing prominent dephosphorylation of nuclear and chromatin-associated regulators following CD276 knockdown. This included core components of the Nucleosome Remodelling and Deacetylase (NuRD) complex such as CHD4 (S308, S310), HDAC2 (S394), and MTA1 (S522) (Fig. 5H). Downregulation of CHD4 phosphorylation is consistent with a shift towards a quiescent chromatin state and reduced proliferative capacity [42]. Also, HDAC2-S394 hypo-phosphorylation is associated with impaired deacetylase activity and induction of growth-inhibitory genes such as *CDKN1A/p21* [43], while MTA1-S522 loss has been linked to reduced cellular adaptability to stress [44]. CD276 knockdown also led to widespread dephosphorylation of cytoskeletal effectors controlling intercellular junctions and migratory behavior. These included CTTN, MYH10, and LMO7, which are key regulators of lamellipodia dynamics and microtubule remodelling [45–47]. The decreased phosphorylation of these proteins suggests a loss of invasive plasticity upon CD276 depletion (Fig. 5H). We further observed phosphosite losses for RB1 and JUN (S63), both of which are known to be critical for cell cycle progression and apoptosis resistance [48, 49]. Conversely, MAPK3 (ERK1) phosphorylation was sustained or elevated, likely reflecting compensatory ErbB signalling in response to CD276 silencing. However, the net signalling effect appears to favour cell cycle arrest and reduced invasion. Collectively, these findings support a role for immature CD276 glycosylation in sustaining kinase-driven signalling programs that promote proliferation, cytoskeletal plasticity, and resistance to apoptosis. Accordingly, its silencing triggered a coordinated shutdown of these pathways, leading to reduced cell cycle activity, reinforcement of junctional architecture, and chromatin stabilization. In contrast, control cells expressing immaturely glycosylated CD276 retain phosphoproteomic signatures of checkpoint adaptation, alternative splicing, and EMT-like transitions, underscoring the role of CD276 as a regulator of invasive reprogramming in colorectal cancer.

Collectively, we have highlighted that CD276 immature glycosylation enhances its oncogenic signalling capacity, promoting CRC cell proliferation, invasion, and resistance to stress through kinase-driven phosphoproteomic reprogramming.

### Aberrantly glycosylated CD276 promotes immune evasion in CRC

**Fig. 6:**
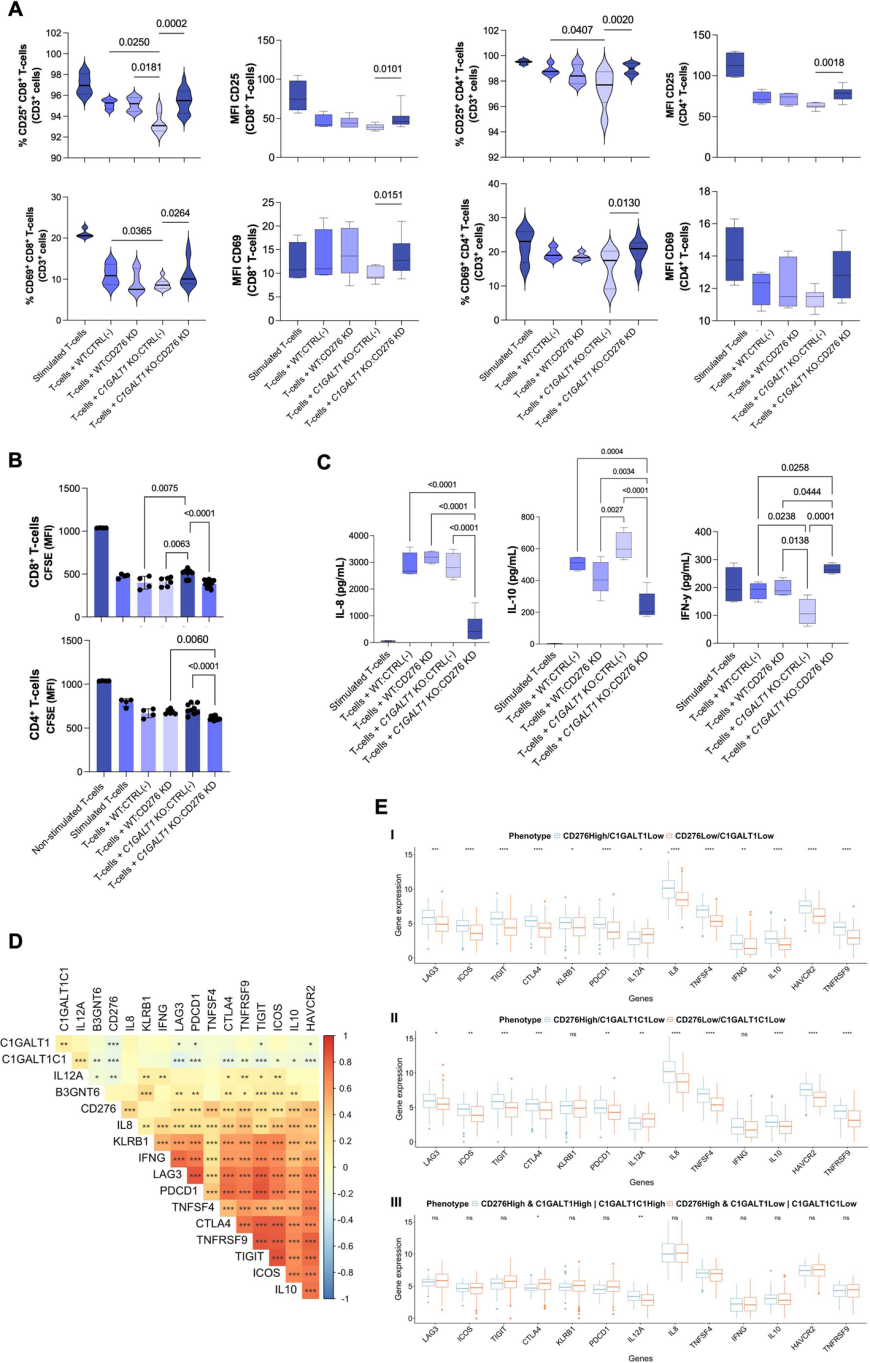
Aberrant CD276 glycosylation suppresses T cell function and shapes an immunosuppressive tumor microenvironment. **A-C** Aberrant CD276 glycosylation dampens T cell activation, proliferation, and functional cytokine output. In vitro co-culture assays and cytokine profiling were conducted using SW620 colorectal cancer cells glycoengineered with *C1GALT1* KO (resulting in immature CD276-Tn glycoforms) and wild-type controls, with or without *CD276* knockdown. These cell models were co-cultured with pre-activated human T cells to evaluate functional immune responses. Across three independent replicates, T cell activation, proliferation, and cytokine secretion were significantly influenced by the glycosylation status of CD276. Statistical comparisons were performed using one-way ANOVA followed by Tukey’s multiple comparisons test. **A** Aberrant CD276 glycosylation dampens T cell activation. Flow cytometry revealed a significant reduction in CD25⁺ cells within both CD4⁺ and CD8⁺ T cell subsets after 5 days of co-culture with *C1GALT1* KO cells. Mean fluorescence intensity (MFI) of CD25 was also lower in these T cells, indicating a subdued activation profile. CD276 knockdown in the KO background restored CD25 expression (both percentage and MFI), implicating glycosylated CD276 in the suppressive phenotype. Similarly, both percentage of CD69⁺ cells and MFI were reduced in T cells after exposure to CD276-expressing KO cells, further supporting impaired activation. Kruskal-Wallis test followed by Dunn’s multiple comparisons test. **B** Abnormal CD276 glycosylation inhibits T cell proliferation. CFSE dilution assays showed higher fluorescence retention in both CD4⁺ and CD8⁺ T cells co-cultured with *C1GALT1* KO cells, indicating reduced proliferation. Silencing CD276 rescued CFSE dilution, confirming that the glycosylated form of CD276 actively suppresses T cell proliferative responses. Kruskal-Wallis test followed by Dunn’s multiple comparisons test. **C** Abnormal CD276 glycosylation reprograms cytokine secretion toward an immunosuppressive profile. Supernatants from co-cultures with *C1GALT1* KO cells displayed elevated IL-8 and IL-10 and reduced IFN-γ levels, compared to *C1GALT1* KO *CD276* KD cells, supporting a shift toward immune suppression. Kruskal-Wallis test followed by Dunn’s multiple comparisons test. **D** Expression correlations suggest an association between CD276, core 1 *O*-glycosylation genes, and immune regulatory markers in patient samples. A correlogram of TCGA CRC samples revealed strong positive correlations between CD276 and immune checkpoint molecules associated with T cell exhaustion, including *PDCD1* (PD-1) and *HAVCR2* (TIM-3). Additionally, *CD276* expression was inversely correlated with *C1GALT1* and *C1GALT1C1 *expression, further suggesting that impaired core 1 *O*-glycosylation may be linked to increased CD276 expression and transcriptional profiles consistent with immune evasion. **E** Tumors co-expressing high levels of *CD276* and low levels of core 1 *O*-glycosylation enzymes exhibit transcriptional profiles indicative of T cell dysfunction and immune suppression. Analysis of TCGA CRC samples revealed that tumors with high *CD276* and low *C1GALT1* expression (Panel I) presented a marked increase in genes associated with T cell exhaustion and an elevation of immunosuppressive cytokines *IL10* and *IL8*, closely mirroring the phenotypes observed in in vitro co-culture assays. Panel II reinforces this observation, showing that tumors with *CD276*^High^/*C1GALT1C1*^Low^ expression recapitulate the immunosuppressive phenotype. Panel III extends this observation by stratifying tumors co-expressing *CD276*^High^ with low expression of both *C1GALT1* and its chaperone *C1GALT1C1* (with high probability of expressing high levels of immature glycosylation), confirming a broader immunosuppressive transcriptional programme that includes increased expression of exhaustion markers such as *CTLA4* and reduced expression of the pro-inflammatory cytokine IL12A. ns = not significant; * = *p* < 0.05; ** = *p* < 0.01; *** = *p* < 0.001; **** = *p* < 0.0001

To assess whether CD276 glycosylation modulates immune regulation, we co-cultured pre-activated, CFSE-labelled human T cells with SW620 wild-type or *C1GALT1*-knockout cells, with or without CD276 knockdown. Flow cytometry showed that T cells co-cultured with *C1GALT1*-KO cells proliferated significantly less, reflected by higher CFSE intensity in both CD4⁺ and CD8⁺ subsets (Fig. 6B). This was accompanied by a marked reduction in early (CD69) and late (CD25) activation markers across both CD4^+^ and CD8^+^ T cell subsets (Fig. 6A, Figure S9). Notably, these immunosuppressive effects were reversed upon CD276 knockdown in the *C1GALT1*-KO background, strongly implicating aberrantly glycosylated CD276 as a key mediator of T cell inhibition.

Furthermore, T cells co-cultured with *C1GALT1* KO cells exhibited a cytokine profile skewed toward an immunosuppressive and regulatory phenotype. This included significantly elevated levels of IL-10 and IL-8 and a corresponding decrease in IFN-γ (Fig. 6C and Figure S10). Given that IL-8 and IL-10 are not canonical T cell cytokines, their elevation may also originate from cancer cells and reflect a contact-dependent response to T cell engagement modulated by the glycosylation status. Broad-spectrum cytokine profiling also revealed a consistent increase in pro-inflammatory mediators, including IL-6, IL-23, IL-17A, and TNF-α, which can either stimulate immune surveillance or drive tumor-promoting inflammation. Notably, elevated IL-23 and IL-17A levels in CRC have been frequently linked to tumor growth and angiogenesis [50]. This is accompanied by increased levels of type 2 and tolerogenic cytokines, such as IL-13 and IL-5 (Figure S10), indicating a shift away from Th1/effector polarization toward a more immune-modulatory state. Importantly, these cytokine alterations were largely reversed upon CD276 knockdown in the *C1GALT1* KO background, restoring IFN-γ production and attenuating IL-10 and IL-8 secretion (Fig. 6C). Together, these findings support a model in which immature CD276 glycosylation promotes immune escape by shaping a non-permissive cytokine environment. Although T cells were pre-activated, direct contact with *C1GALT1* KO cells in co-culture suppressed their activation and prevented them from acquiring effector functions. This observation aligns with previous reports showing that cancer cells can respond to T cell–derived inflammatory cues such as TNF-α, via NF-κB activation, by increasing IL-8 production. IL-8 is a chemokine that recruits MDSCs and TAMs, promotes angiogenesis, and reinforces immune exclusion [51]. In parallel, IL-10 suppresses T cell function and promotes peripheral tolerance [52]. In addition, reduced IFN-γ reflects impaired effector polarization of CD4⁺ T cells and reduced cytotoxic capacity of CD8 ^+^ T cells [53]. In summary, our data define a glycosylation-dependent mechanism of immune modulation. The acquisition of immature glycosylation by CD276 in cancer cells contributes to the establishment of a suppressive cytokine milieu and an immune-excluded, tumor-permissive niche.

We then interrogated TCGA CRC datasets to find support for these findings in clinical samples. Correlation and clustering analyses linked low *C1GALT1*/*C1GALT1C1* expression to higher levels of immune checkpoints and T cell exhaustion markers (Fig. 6D), including PD-1 (*PDCD1*) and TIM-3 (*HAVCR2*) [54]. In contrast, *B3GNT6* expression showed weaker positive correlations with immune-related genes, suggesting a more limited role in modulating immune escape. CD276 expression itself was positively correlated with multiple immune checkpoints such as *PDCD1* and *HAVCR2*, and inversely correlated with *C1GALT1* and *C1GALT1C1* (Fig. 6D), as previously described. To explore this further, we stratified tumors based on *CD276* and *C1GALT1* expression levels to mirror the in vitro phenotype for CD276 with immature glycosylation. Tumors with high *CD276* and low *C1GALT1* expressions (*CD276*^High^/*C1GALT1*^Low^) exhibited significantly elevated levels of exhaustion markers including *TIGIT*, *LAG3*, *HAVCR2*, and *PDCD1*, when compared to *CD276*^Low^/*C1GALT1*^Low^ tumors (Fig. 6E, Panel I). A similar pattern emerged when stratifying by *C1GALT1C1* expression (*CD276*^High^/*C1GALT1C1*^Low^ vs. *CD276*^Low^/*C1GALT1C1*^Low^; Fig. 6E, Panel II). These observations suggest that deficiencies in core 1 *O*-glycosylation, whether C1GalT1 or its chaperone, potentiate CD276-associated immune evasion. Cytokine gene expression analysis further supported the presence of a reprogrammed immune environment. *IL10* and *IL8* levels were significantly elevated, whereas *IL12A* was reduced in both *CD276*^High^/*C1GALT1*^Low^ and *CD276*^High^/*C1GALT1C1*^Low^ tumors, mirroring cytokine shifts observed in vitro. Interestingly, *IFNG* expression was elevated only in *CD276*^High^/*C1GALT1*^Low^ tumors. This suggests that T cells may become initially activated but subsequently drive toward exhaustion due to sustained immune checkpoint signalling, which warrants future demonstration. In contrast, when CD276 remained highly expressed, differences in *C1GALT1*/*C1GALT1C1* levels no longer affected most immune-related genes, except for CTLA4 and IL12A (Fig. 6E, Panel III). Collectively, our results indicate that CD276 is a strong driver of immunosuppression, an effect amplified by impaired O-glycosylation.

## Discussion

The glycome is pivotal in cancer progression and dissemination, offering significant potential for the precise detection, targeting, and elimination of cancer cells. Despite recent advances in understanding CRC glycome alterations by high-throughput mass spectrometry, a considerable gap in grasping the underlying functional and clinical implications of these alterations remains [37, 55, 56]. This study addresses these gaps through an in-depth exploration of *O*-glycosylation and its impact in CRC.

We confirmed previous findings linking premature termination of *O*-glycan elongation to cancer aggressiveness [57]. We began by identifying glycogene deregulation in colorectal tumors compared to normal tissue, with *B3GNT6* downregulation emerging as a poor prognosis marker in CRC. When accompanied by concomitant downregulation of *C1GALT1* and/or *C1GALT1C1*, this alteration was also significantly associated with decreased overall survival in patients. Advancing beyond current knowledge, we demonstrate that these events coincide with the loss of normal colonic glycosylation. This was predominantly characterized by the loss of *O*−6 sialylated core 3 structures, likely supported by *B3GNT6* and *ST6GALNACs* expression in healthy tissues. Moreover, we found that advanced epithelial-like and mesenchymal-like CRC showed distinct glycocode profiles. Epithelial-like tumors displayed an intermediate glycome between normal colon tissue and mesenchymal-like cancers. In these cases, the loss of core 3-related structures was not compensated by the capacity to shift the glycophenotype toward core 1 and core 2-related structures, resulting in an enrichment for short Tn and sTn antigens. Mesenchymal-like tumors, while also exhibiting high levels of Tn and sTn antigens, were predominantly enriched for sialylated T antigens. Interestingly, tumors of both differentiation states that showed signs of distant metastases also displayed extended core 2 structures, including glycans compatible with the presence of sLe antigens. These antigens are known to facilitate cancer cell intravasation into the bloodstream and colonization of distant locations by mediating adhesion to endothelial cells via E-selectin [58]. Such findings reinforce previous reports and underscore the heterogeneous and dynamic nature of the CRC glycome, where distinct subpopulations may coexist to drive aggressive traits [56, 59]. We further broadened our understanding of the events potentially dictating immature glycosylation at the surface of CRC cells. Here, we show that in more aggressive tumors associated with an unfavourable prognosis, the concurrent downregulation of *B3GNT6* and *C1GALT1* plays a decisive role in driving immature glycosylation. Interestingly, this has previously been largely attributed to *C1GALT1C1* loss-of-function mutations [60]. Utilizing *C1GALT1* knockout cell lines with distinct molecular backgrounds, we confirmed that immature glycosylation drives enhanced invasion and, in a context-dependent manner, increases proliferation. This reinforces previous observations regarding *C1GALT1C1* and decisively links the acquisition of immature glycophenotypes through different mechanisms to cancer aggressiveness [61].

We further identified CD276 as a carrier of immature *O*-glycans in CRC. CD276, also known as B7-H3, belongs to the B7 family of immune checkpoint molecules and can either enhance or suppress immune responses depending on context [62]. Specifically, it has been shown to interact with cognate receptors on immune cells, particularly T cells, regulating their activation and function [62]. CD276 is overexpressed in several malignancies, including CRC [11], where its expression correlates with poor prognosis. Our findings go beyond the state-of-the-art by demonstrating that immature *O*-glycosylation also regulates CD276 stability, enhances its expression, and shapes its immunomodulatory role in CRC. This underscores that CD276 regulation in cancer is not solely governed by N-glycans but is also critically shaped by the *O*-glycome, revealing new layers of regulatory control and therapeutic potential. Site-specific N-glycosylation was recently shown to stabilise CD276, holding therapeutic potential [63]. In addition, FUT8-mediated core fucosylation has been implicated in promoting CD276-dependent immune escape in triple-negative breast cancer [15]. Together, these observations highlight a growing paradigm in which distinct glycan classes tune CD276 biology. Within this emerging framework, our study now identifies a distinct and previously unrecognised regulatory axis driven by immature *O*-glycans. Specifically, we found that the loss of core-1 *O*-glycan extension leading to immature glycosylation promotes CD276 accumulation and functional activity. These are novel observations, supporting that this immune checkpoint is subject to dual glycosylation control at both the *N*- and *O*-glycan levels. Notably, CD276 aberrant *O*-glycoforms were abundant across primary tumours and metastases but absent from healthy colon, denoting a cancer-specific modification with clear translational relevance. Moreover, expression of CD276 carrying immature *O*-glycans was enriched in right-sided tumors, which are commonly associated with more aggressive clinical behavior. It was also linked to significantly worse patient survival. Nevertheless, proteomic and glycoproteomic analyses further show that CD276 is broadly expressed across tumor stages and differentiation states. However, the density and distribution of its glycosylation sites vary with tumor differentiation, with potential implications for tumor behavior and therapeutic design. CID-based MS/MS analyses support this, indicating that specific* O*-glycosylation sites may be differentially occupied in epithelial-like versus mesenchymal-like tumors. In fact, CID spectra provide strong peptide backbone coverage and consistent glycan neutral-loss patterns, offering robust evidence for immature *O*-glycans on CD276. Nevertheless, because CID cannot fully localize *O*-glycosylation sites, these positional differences remain tentative. Even so, the emerging pattern of subtype-dependent glycosylation reinforces the idea that CD276 glycoforms encode additional layers of tumor biology that remain unexplored. Definitive confirmation will require electron-based dissociation methods such as ETD or EThcD, which preserve glycan–peptide linkages and are essential for resolving site-specific *O*-glycosylation. Beyond this, a more refined glycoproteomic workflow will be needed. This should include robust glycopeptide enrichment and functional assays to determine how specific CD276 glycoforms influence protein stability, receptor interactions, and immune-evasion pathways. Such resolution will be critical to refine CD276-targeted strategies by accounting for glycoform diversity that may affect antibody recognition and ligand binding.

We also show that loss or downregulation of *C1GALT1* promotes immature *O*-glycosylation of CD276, leading to increased protein stability and elevated expression. Although our data support a posttranslational stabilization mechanism, the basis for the observed transcriptional upregulation of CD276 remains unclear. One plausible explanation is that defective *O*-glycosylation perturbs cellular homeostasis, activating compensatory stress response pathways. Previous studies have shown that glycosylation defects can induce endoplasmic reticulum stress or Golgi dysfunction, triggering transcriptional programs that upregulate membrane protein expression [64, 65]. CD276 may therefore be indirectly upregulated as part of a broader adaptive response, which warrants further exploration. Interestingly, emerging evidence suggests that glycosylation status can modulate epigenetic and transcriptional regulators. Altered glycosylation has been shown to affect the stability and function of key transcription factors, such as SP1 and STAT3, both of which are implicated in immune checkpoint regulation [66, 67]. It is conceivable that similar mechanisms contribute to increased CD276 transcription in CRC. Although the precise pathways remain to be defined, our findings lay the groundwork for future investigations into how glycome remodelling interfaces with CD276 transcriptional control in cancer.

Functionally, we demonstrated that immature glycosylation supports CD276-mediated invasion through phospho-signalling rewiring, enhances cell proliferation, and suppresses T cell activation. In vitro, *CD276* knockdown in glycoengineered cells reduced both invasive capacity and proliferation, particularly in metastatic models. Quantitative phosphoproteomic analysis revealed that aberrantly glycosylated CD276 sustains pro-invasive and pro-proliferative signalling through kinases such as CSNK2A2, AKT1, and CDK2 and modulates chromatin and cytoskeletal regulators, including HDAC2, CHD4, and cortactin [38–40, 68, 69]. These findings define a cell-intrinsic function of CD276, wherein its abnormal glycosylation promotes tumor aggressiveness through the activation of oncogenic signalling cascades. Among the altered phospho-nodes, AKT1, CSNK2A2 and cortactin (CTTN) emerged as central regulators directly linked to the observed invasive and proliferative phenotypes. AKT1 is a master coordinator of cytoskeletal remodelling, focal-adhesion turnover and cell survival [70–72], while CSNK2A2 (CK2) drives actin dynamics, invasion and EMT through phosphorylation of cytoskeletal substrates and adhesion regulators [73, 74]. Increased phosphorylation of CTTN is strongly associated with enhanced Arp2/3-dependent actin branching, lamellipodia formation and metastatic behaviour [75–77]. These nodes therefore provide a mechanistic bridge between CD276-driven signalling and the aggressive phenotypes detected experimentally. In contrast, phospho-changes in chromatin remodelling molecules such as HDAC2 and CHD4, although being also biologically relevant, are less directly connected to acute invasion and proliferation. Instead, they may reflect slower or more global transcriptional adaptations [78–80], warranting deeper investigation.

Aberrant CD276 glycosylation reprogrammed cytokine profiles, promoting IL-10 and IL-8 production while decreasing proinflammatory IFN-γ secretion. These changes suggest that aberrantly glycosylated CD276 not only impairs T cell activation but also actively reconditions the tumor microenvironment to favour immune evasion [51, 52, 81, 82]. Two non–mutually exclusive mechanisms may explain how immature CD276 glycosylation suppresses T cell function. We hypothesize that CD276-Tn/sTn may act as a ligand for yet-unidentified immune receptors, with truncated *O*-glycans potentially modulating affinity for lectin-type receptors expressed on T cells. Among plausible candidates are inhibitory Siglec family members such as Siglec-7 and Siglec-9, which are present on activated and exhausted T cell subsets and transmit ITIM-mediated suppressive signals [83, 84]. Engagement of such receptors by CD276-Tn/sTn could therefore represent a potential route for attenuating T cell activation and effector function. Building on these findings, we are actively pursuing the identification of CD276-binding receptors using a combination of proteomics-assisted ligand-binding assays and unbiased receptor-profiling strategies. On the other hand, phospho-signaling rewiring associated with altered CD276 glycosylation may help explain the shift in cytokine secretion toward an immunosuppressive niche. AKT1, CSNK2A2, CDK2 and chromatin regulators including HDAC2 and CHD4, all found differentially phosphorylated in our dataset, have previously been linked to IL-10 and IL-8 induction as well as to the suppression of IFN-γ-driven responses, providing a mechanistic rationale consistent with the cytokine profile observed here [85–88]. However, because cytokine measurements were obtained from tumour–T cell co-cultures, our data do not allow attribution of these changes to tumour cells or T cells individually. Taken together, both receptor-mediated interactions and oncogenic signaling-driven cytokine modulation remain plausible and may even act synergistically to impair T cell activity. Future work should dissect their relative contributions, for example by comparing cytokine production in tumour monocultures versus tumour–T-cell co-cultures and by perturbing candidate signaling pathways.

In summary, we position immaturely glycosylated CD276 as a multifunctional effector that coordinates tumor-intrinsic signalling and immune suppression. By enhancing pro-invasive and pro-proliferative signalling while dampening T cell activation, aberrant CD276 *O*-glycosylation grants cancer cells a dual advantage that fuels both aggressive growth and immune evasion. This mechanistic framework links glycosylation-driven CD276 remodelling to key hallmarks of colorectal cancer progression. Overall, we propose that CD276 *O*-glycosylation remodelling is a novel driver of immune checkpoint activity and tumor aggressiveness in CRC. Future work should validate these mechanisms in vivo. It should also clarify how other CRC-associated glycans, including extended core 2 structures linked to metastasis [37], influence CD276 function. Understanding the impact of CD276 on other immune cell types will also be essential for designing appropriate countermeasures. From a translational perspective, our results identify immaturely glycosylated CD276 as a promising therapeutic target. CD276 and *C1GALT1/C1GALT1C1* transcript levels also provide a simple basis for patient stratification and may help identify those more likely to benefit from future CD276 glyco-targeted therapies. These findings are consistent with recent efforts to exploit *N*-glycan–dependent regulation of CD276 [63]. They also suggest that different glycan-defined states of CD276 may support distinct precision-targeting strategies. Therefore, this is also an opportunity to improve on strategies targeting CD276 with antibodies [89], antibody–drug conjugates [90, 91] and cellular therapies, including several CD276-CAR-T cell products [92–94]. Therapies engineered to recognise the *O*-glycan–dependent epitope, rather than total CD276, may achieve greater tumour specificity and reduce on-target/off-tumour toxicity. Notably, glycans themselves are increasingly being pursued as therapeutic entry points, with tumour-associated carbohydrate antigens already targeted by glycan-specific CAR-T cells [95, 96], monoclonal antibodies [97, 98], ADCs [99, 100] and glycoconjugate vaccines [101–103]. Individually, both CD276-directed and glycan-focused interventions are already showing translational promise but may be challenged by insufficient cancer specificity. Integrating these two dimensions by incorporating glycan specificity into CD276-targeted modalities, particularly towards CD276-Tn/sTn combotopes, may ultimately yield a new generation of highly selective and clinically more effective immunotherapies. Such an approach will nevertheless require careful characterization and management of glycan microheterogeneity and rigorous assessment of potential cross-reactivity with other Tn/sTn-bearing proteins. Collectively, we argue that that glycan-resolved targeting of CD276, potentially integrating both *N*- and *O*-glycans–defined disease states, represents a rational blueprint for the next generation of precision immunotherapies.

## Conclusion

This work provides foundational insights for the rational design of therapeutic strategies that selectively target aberrantly glycosylated CD276 in cancer. Together, our findings reveal a previously unrecognized link between cancer-associated glycome remodelling and immune evasion in colorectal cancer. By suggesting that CD276 is a glycosylation-dependent immune checkpoint, this study opens new avenues for innovative therapeutic strategies aimed at selectively targeting tumor-specific glycoforms. Future efforts to characterize the broader glycocode of the tumor microenvironment, including the identification of receptors and lectin networks that interpret this altered glycosylation landscape. This will be critical to unlocking next-generation immunotherapies and advancing precision oncology.

## Supplementary Information

Supplementary Material 1.

Supplementary Material 2.

Supplementary Material 3.

Supplementary Material 4.

Supplementary Material 5.

## Data Availability

All the data generated or analysed during this study are included in this published article and its supplementary information files. Glycomics data have been deposited at GlycoPost (accession number GPST000606, project: GPST000606.0) [104].

## Abbreviations

ATCC: American Type Culture Collection
CHSJ: Centro Hospitalar São João
CMS: Consensus Molecular Subtypes;
CORA: Cellular O-Glycome Reporter/Amplification
CRC: Colorectal Cancer
dsT: Disialyl-T
DTT: Dithiothreitol
EMT: Epithelial–Mesenchymal Transition
FBS: Fetal Bovine Serum
FC: Flow Cytometry
FDR: False Discovery Rate
FFPE: Formalin-Fixed Paraffin-Embedded
FMO: Fluorescence Minus One
HCD: Higher-Energy Collisional Dissociation
HR: Hazard Ratio
ICIs: Immune Checkpoint Inhibitors
IDAA: Indel Detection by Amplicon Analysis
IF: Immunofluorescence
IgC: Immunoglobulin Constant Domain
IgV: Immunoglobulin Variable Domain
IHC: Immunohistochemistry
IP: Immunoprecipitation
IPO Porto: Portuguese Institute of Oncology of Porto
KO: Knockout
KSEA: Kinase-Substrate Enrichment Analysis
MFI: Mean Fluorescence Intensity
MS: Mass Spectrometry
MSI: Microsatellite Instability
MSS: Microsatellite Stability
NeuAse: Neuraminidase
NSAF: Normalized Spectral Abundance Factor
OS: Overall Survival
PCA: Principal Component Analysis
