# Supplementary figures and images for: CD276 immature glycosylation drives colorectal cancer aggressiveness and T cell mediated immune escape
Source: Cell Commun Signal. 2026 Jan 20;24:113. doi: 10.1186/s12964-026-02672-y (PMC12903257; doi:10.1186/s12964-026-02672-y)

UNCROPPED BLOTS FOR FIGURE 4

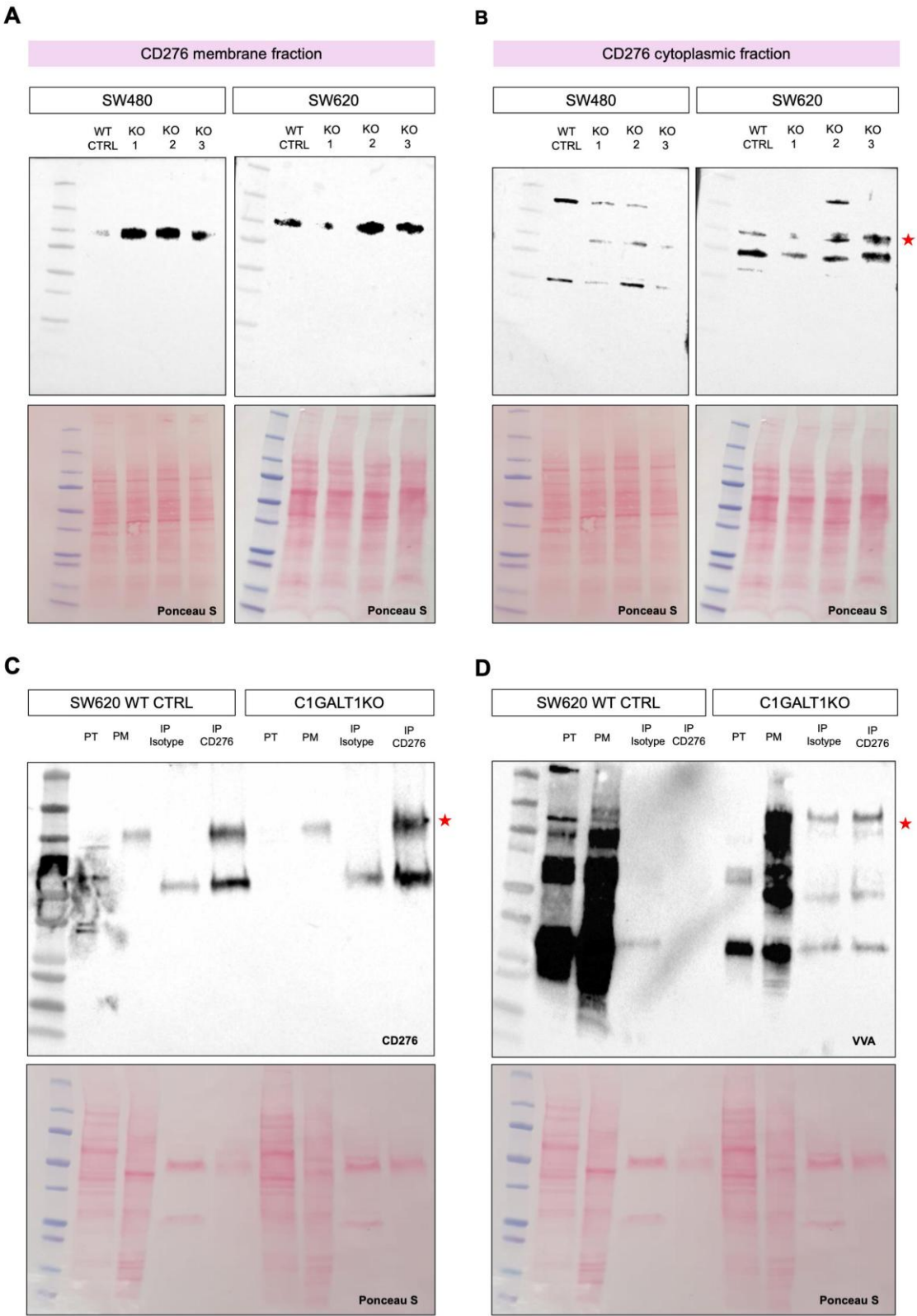

D

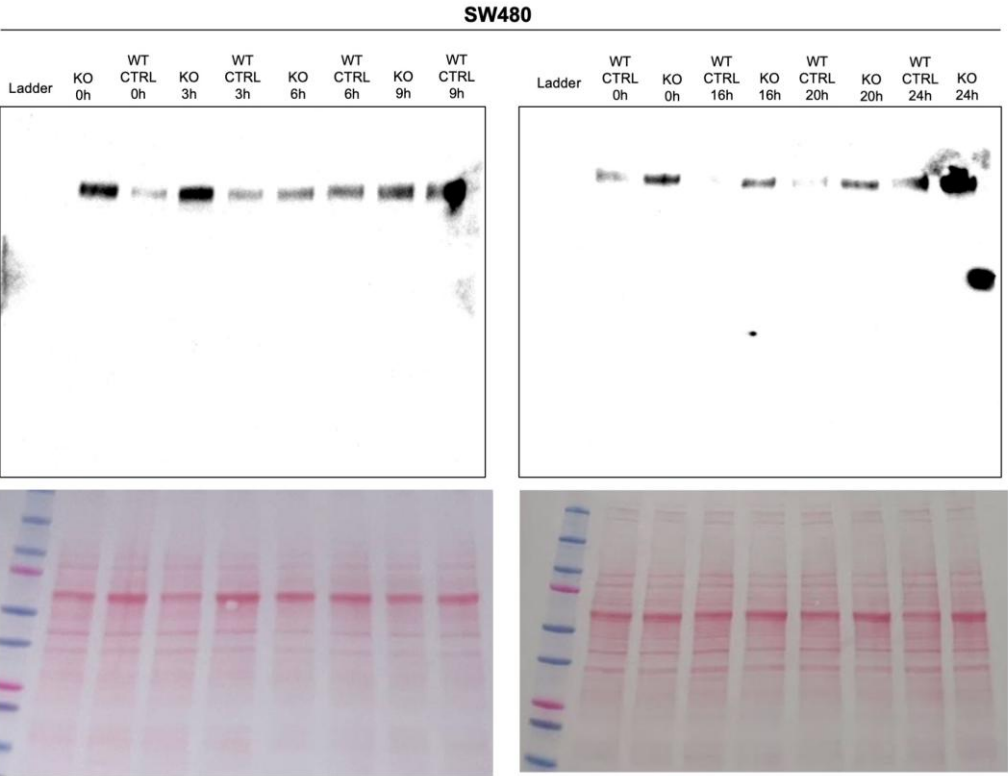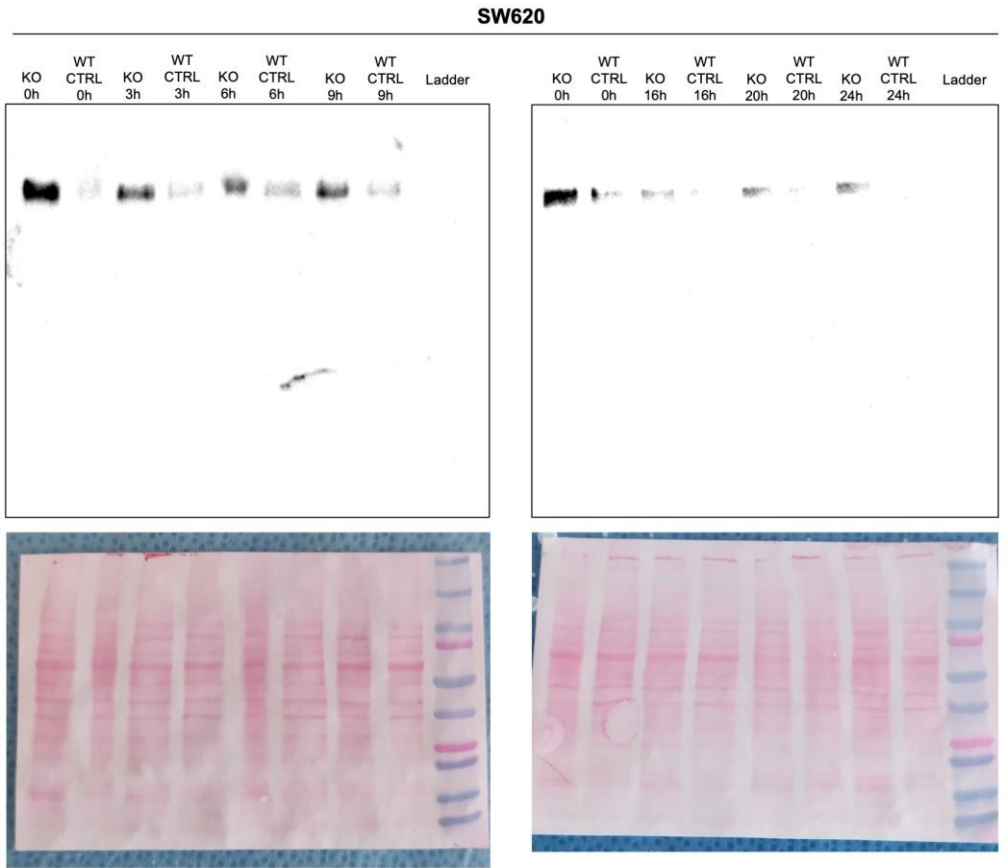

Supplement: Supplementary file 4 — Supplementary Material 4. [file 12964_2026_2672_MOESM4_ESM.pdf]
