# Supplementary material for: CD276 immature glycosylation drives colorectal cancer aggressiveness and T cell mediated immune escape
Source: Cell Commun Signal. 2026 Jan 20;24:113. doi: 10.1186/s12964-026-02672-y (PMC12903257; doi:10.1186/s12964-026-02672-y)
Supplement: Supplementary file 5 — Supplementary Material 5. [file 12964_2026_2672_MOESM5_ESM.zip › supplementary data file 2.pdf]

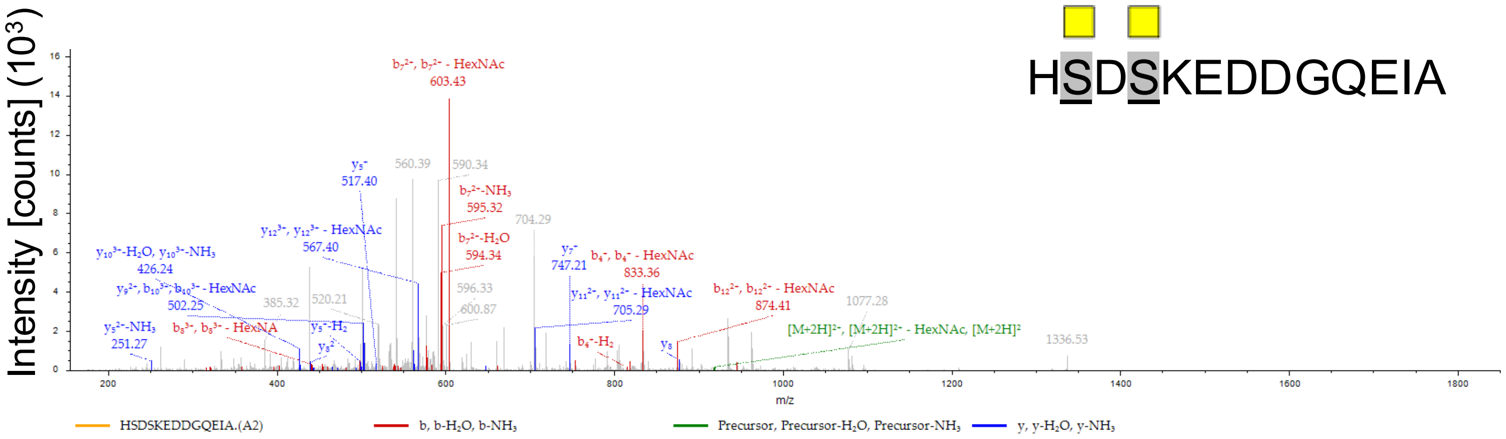

#1	b⁺	b²⁺	b³⁺	Seq.	y⁺	y²⁺	y³⁺	#2
1	138.06619	69.53673	46.69358	H				13
2	428.17759	214.59243	143.39738	S-HexNAc	1699.70284	850.35506	567.23913	12
3	543.20453	272.10590	181.73970	D	1409.59144	705.29936	470.53533	11
4	833.31593	417.16161	278.44350	S-HexNAc	1294.56450	647.78589	432.19302	10
5	961.41090	481.20909	321.14182	K	1004.45309	502.73019	335.48922	9
6	1090.45349	545.73038	364.15601	E	876.35813	438.68270	292.79090	8
7	1205.48043	603.24385	402.49833	D	747.31554	374.16141	249.77670	7
8	1320.50738	660.75733	440.84064	D	632.28860	316.64794	211.43438	6
9	1377.52884	689.26806	459.84780	G	517.26165	259.13446	173.09207	5
10	1505.58742	753.29735	502.53399	Q	460.24019	230.62373	154.08491	4
11	1634.63001	817.81864	545.54819	E	332.18161	166.59444	111.39872	3
12	1747.71407	874.36068	583.24288	I	203.13902	102.07315	68.38452	2
13				A	90.05496	45.53112	30.68984	1

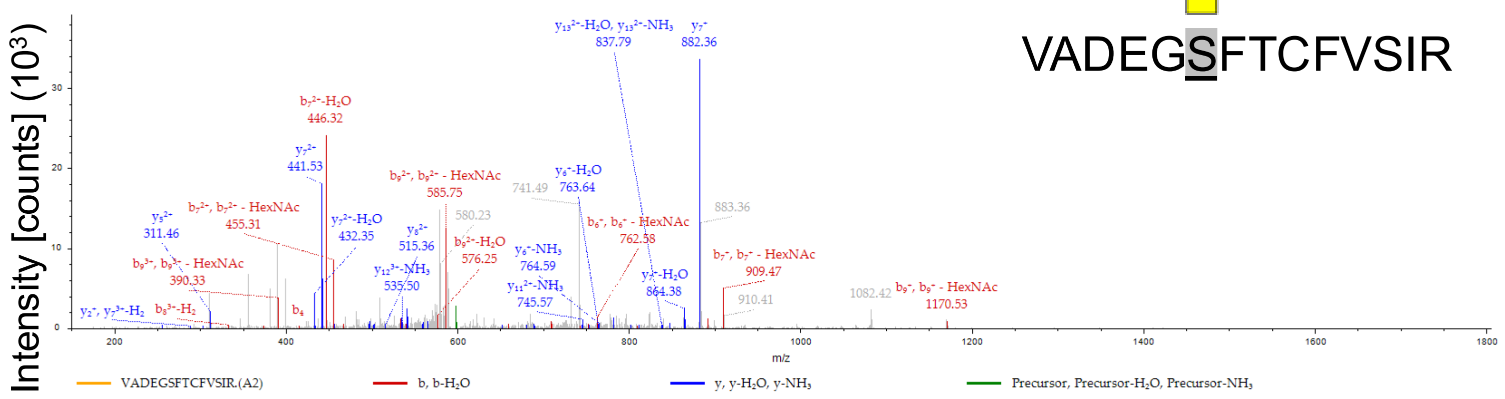

VADEGSFTCFVSIR

#1	b⁺	b²⁺	b³⁺	Seq.	y⁺	y²⁺	y³⁺	#2
1	100.07569	50.54148	34.03008	V				14
2	171.11280	86.06004	57.70912	A	1691.75813	846.38270	564.59089	13
3	286.13975	143.57351	96.05143	D	1620.72101	810.86415	540.91186	12
4	415.18234	208.09481	139.06563	E	1505.69407	753.35067	502.56954	11
5	472.20380	236.60554	158.07279	G	1376.65148	688.82938	459.55534	10
6	762.31521	381.66124	254.77659	S-HexNAc	1319.63001	660.31865	440.54819	9
7	909.38362	455.19545	303.79939	F	1029.51861	515.26294	343.84439	8
8	1010.43130	505.71929	337.48195	T	882.45020	441.72874	294.82158	7
9	1170.46195	585.73461	390.82550	-Carbamidometh	781.40252	391.20490	261.13902	6
10	1317.53036	659.26882	439.84830	F	621.37187	311.18957	207.79548	5
11	1416.59877	708.80303	472.87111	V	474.30346	237.65537	158.77267	4
12	1503.63080	752.31904	501.88179	S	375.23504	188.12116	125.74987	3
13	1616.71487	808.86107	539.57647	I	288.20302	144.60515	96.73919	2
14				R	175.11895	88.06311	59.04450	1

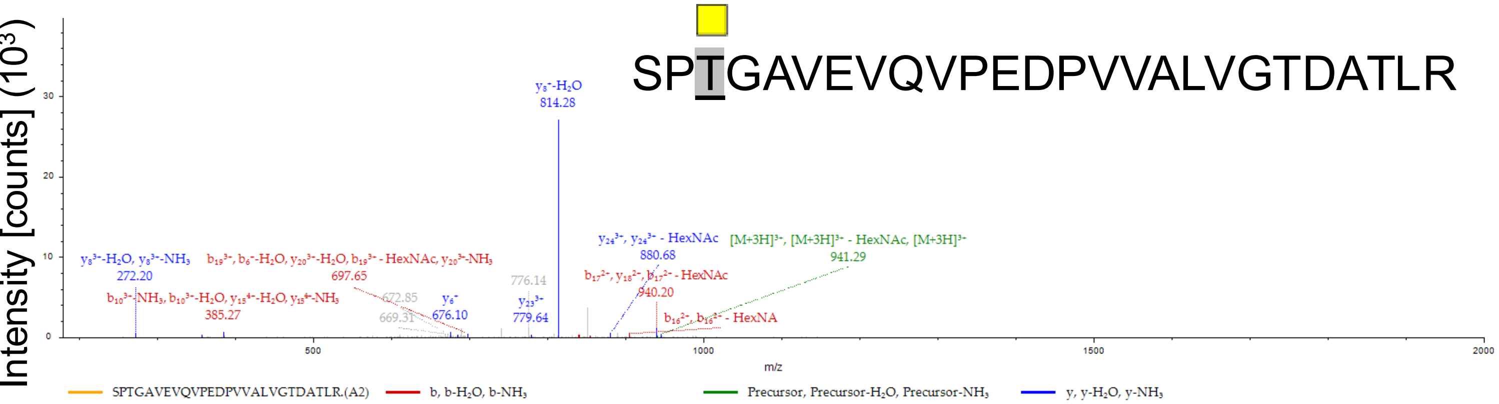

#1	b⁺	b²⁺	b³⁺	b⁴⁺	Seq.	y⁺	y²⁺	y³⁺	y⁴⁺	#2
1	88.03930	44.52329	30.01795	22.76528	S					26
2	185.09207	93.04967	62.36887	47.02847	P	2736.43018	1368.71873	912.81491	684.86300	25
3	489.21912	245.11320	163.74456	123.06024	T-HexNAc	2639.37742	1320.19235	880.46399	660.59981	24
4	546.24058	273.62393	182.75171	137.31560	G	2335.25037	1168.12882	779.08831	584.56805	23
5	617.27770	309.14249	206.43075	155.07488	A	2278.22890	1139.61809	760.08115	570.31268	22
6	716.34611	358.67669	239.45355	179.84199	V	2207.19179	1104.09953	736.40211	552.55340	21
7	845.38870	423.19799	282.46775	212.10263	E	2108.12337	1054.56533	703.37931	527.78630	20
8	944.45712	472.73220	315.49056	236.86974	V	1979.08078	990.04403	660.36511	495.52565	19
9	1072.51570	536.76149	358.17675	268.88438	Q	1880.01237	940.50982	627.34231	470.75855	18
10	1171.58411	586.29569	391.19955	293.65148	V	1751.95379	876.48053	584.65611	438.74391	17
11	1268.63687	634.82208	423.55048	317.91468	P	1652.88538	826.94633	551.63331	413.97680	16
12	1397.67947	699.34337	466.56467	350.17532	E	1555.83261	778.41994	519.28239	389.71361	15
13	1512.70641	756.85684	504.90699	378.93206	D	1426.79002	713.89865	476.26819	357.45296	14
14	1609.75917	805.38322	537.25791	403.19525	P	1311.76308	656.38518	437.92588	328.69623	13
15	1708.82759	854.91743	570.28071	427.96235	V	1214.71031	607.85879	405.57496	304.43304	12
16	1807.89600	904.45164	603.30352	452.72946	V	1115.64190	558.32459	372.55215	279.66593	11
17	1878.93311	939.97020	626.98256	470.48874	A	1016.57348	508.79038	339.52935	254.89883	10
18	1992.01718	996.51223	664.67724	498.75975	L	945.53637	473.27182	315.85031	237.13955	9
19	2091.08559	1046.04643	697.70005	523.52686	V	832.45231	416.72979	278.15562	208.86853	8
20	2148.10706	1074.55717	716.70720	537.78222	G	733.38389	367.19559	245.13282	184.10143	7
21	2249.15473	1125.08101	750.38976	563.04414	T	676.36243	338.68485	226.12566	169.84606	6
22	2364.18168	1182.59448	788.73208	591.80088	D	575.31475	288.16101	192.44310	144.58415	5
23	2435.21879	1218.11303	812.41111	609.56016	A	460.28781	230.64754	154.10079	115.82741	4
24	2536.26647	1268.63687	846.09367	634.82207	T	389.25069	195.12899	130.42175	98.06813	3
25	2649.35053	1325.17891	883.78836	663.09309	L	288.20302	144.60515	96.73919	72.80621	2
26					R	175.11895	88.06311	59.04450	44.53520	1

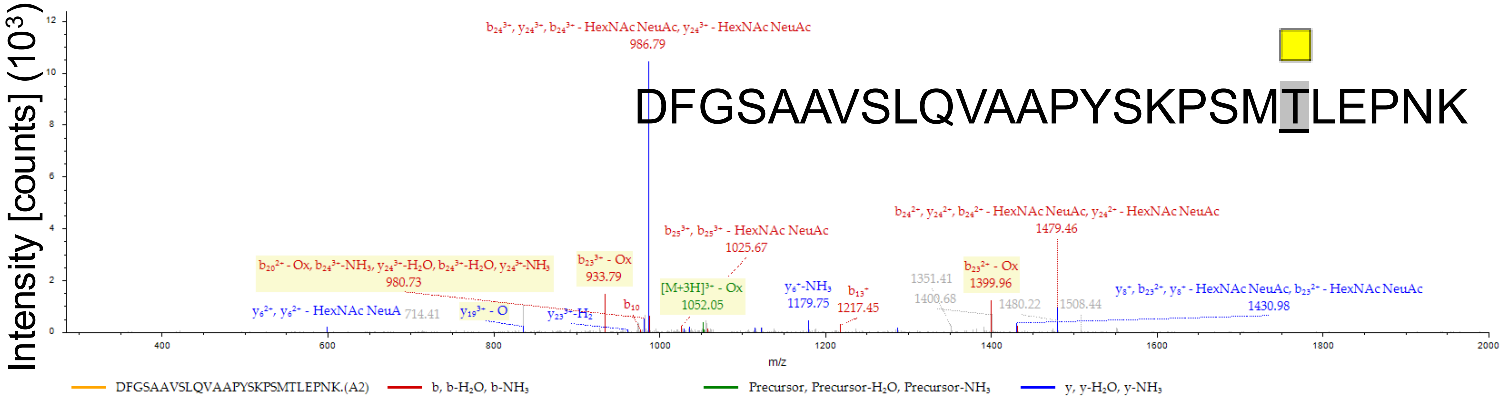

#1	b^+	b^{2+}	b^{3+}	b^{4+}	Seq.	y^+	y^{2+}	y^{3+}	y^{4+}	#2
1	116.03422	58.52075	39.34959	29.76401	D					26
2	263.10263	132.05496	88.37240	66.53112	F	3015.48671	1508.24699	1005.83375	754.62714	25
3	320.12410	160.56569	107.37955	80.78648	G	2868.41830	1434.71279	956.81095	717.86003	24
4	407.15613	204.08170	136.39023	102.54449	S	2811.39683	1406.20205	937.80380	703.60467	23
5	478.19324	239.60026	160.06926	120.30377	A	2724.36480	1362.68604	908.79312	681.84666	22
6	549.23035	275.11881	183.74830	138.06305	A	2653.32769	1327.16748	885.11408	664.08738	21
7	648.29877	324.65302	216.77111	162.83015	V	2582.29058	1291.64893	861.43504	646.32810	20
8	735.33080	368.16904	245.78178	184.58816	S	2483.22216	1242.11472	828.41224	621.56100	19
9	848.41486	424.71107	283.47647	212.85917	L	2396.19013	1198.59871	799.40156	599.80299	18
10	976.47344	488.74036	326.16266	244.87382	Q	2283.10607	1142.05667	761.70687	571.53198	17
11	1075.54185	538.27456	359.18547	269.64092	V	2155.04749	1078.02739	719.02068	539.51733	16
12	1146.57896	573.79312	382.86451	287.40020	A	2055.97908	1028.49318	685.99788	514.75023	15
13	1217.61608	609.31168	406.54354	305.15948	A	1984.94197	992.97462	662.31884	496.99095	14
14	1314.66884	657.83806	438.89447	329.42267	P	1913.90485	957.45606	638.63980	479.23167	13
15	1477.73217	739.36972	493.24891	370.18850	Y	1816.85209	908.92968	606.28888	454.96848	12
16	1564.76420	782.88574	522.25958	391.94651	S	1653.78876	827.39802	551.93444	414.20265	11
17	1692.85916	846.93322	564.95790	423.97025	K	1566.75673	783.88200	522.92376	392.44464	10
18	1789.91193	895.45960	597.30883	448.23344	P	1438.66177	719.83452	480.22544	360.42090	9
19	2080.02333	1040.51530	694.01263	520.76129	S-HexNAc	1341.60900	671.30814	447.87452	336.15771	8
20	2227.05873	1114.03300	743.02443	557.52014	M-Oxidation	1051.49760	526.25244	351.17072	263.62986	7
21	2531.18578	1266.09653	844.40011	633.55190	T-HexNAc	904.46220	452.73474	302.15892	226.87101	6
22	2644.26984	1322.63856	882.09480	661.82292	L	600.33515	300.67121	200.78324	150.83925	5
23	2773.31243	1387.15986	925.10900	694.08357	E	487.25109	244.12918	163.08855	122.56823	4
24	2870.36520	1435.68624	957.45992	718.34676	P	358.20850	179.60789	120.07435	90.30758	3
25	2984.40813	1492.70770	995.47423	746.85749	N	261.15573	131.08150	87.72343	66.04439	2
26					K	147.11280	74.06004	49.70912	37.53366	1

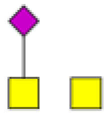

TALQPLHSDSKEDDGQEIA

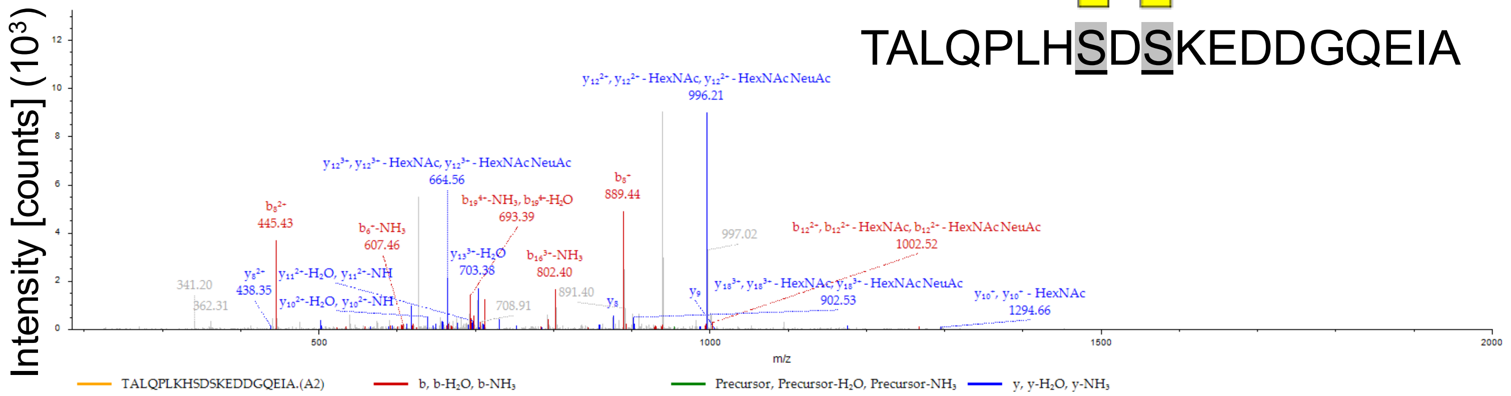

#1	b⁺	b²⁺	b³⁺	b⁴⁺	Seq.	y⁺	y²⁺	y³⁺	y⁴⁺	#2
1	102.05496	51.53112	34.68984	26.26920	T					20
2	173.09207	87.04967	58.36887	44.02847	A	2778.26871	1389.63800	926.76109	695.32264	19
3	286.17613	143.59170	96.06356	72.29949	L	2707.23160	1354.11944	903.08205	677.56336	18
4	414.23471	207.62099	138.74975	104.31414	Q	2594.14754	1297.57741	865.38736	649.29234	17
5	511.28747	256.14738	171.10068	128.57733	P	2466.08896	1233.54812	822.70117	617.27770	16
6	624.37154	312.68941	208.79536	156.84834	L	2369.03620	1185.02174	790.35025	593.01451	15
7	752.46650	376.73689	251.49368	188.87208	K	2255.95213	1128.47970	752.65556	564.74349	14
8	889.52541	445.26634	297.17999	223.13681	H	2127.85717	1064.43222	709.95724	532.71975	13
9	1470.73223	735.86975	490.91559	368.43851	HexNAc(1)NeuAc	1990.79826	995.90277	664.27094	498.45502	12
10	1585.75917	793.38322	529.25791	397.19525	D	1409.59144	705.29936	470.53533	353.15332	11
11	1875.87057	938.43893	625.96171	469.72310	S-HexNAc	1294.56450	647.78589	432.19302	324.39658	10
12	2003.96554	1002.48641	668.66003	501.74684	K	1004.45309	502.73019	335.48922	251.86873	9
13	2133.00813	1067.00770	711.67423	534.00749	E	876.35813	438.68270	292.79090	219.84499	8
14	2248.03507	1124.52117	750.01654	562.76423	D	747.31554	374.16141	249.77670	187.58434	7
15	2363.06202	1182.03465	788.35886	591.52096	D	632.28860	316.64794	211.43438	158.82761	6
16	2420.08348	1210.54538	807.36601	605.77633	G	517.26165	259.13446	173.09207	130.07087	5
17	2548.14206	1274.57467	850.05220	637.79097	Q	460.24019	230.62373	154.08491	115.81550	4
18	2677.18465	1339.09596	893.06640	670.05162	E	332.18161	166.59444	111.39872	83.80086	3
19	2790.26871	1395.63800	930.76109	698.32264	I	203.13902	102.07315	68.38452	51.54021	2
20					A	90.05496	45.53112	30.68984	23.26920	1

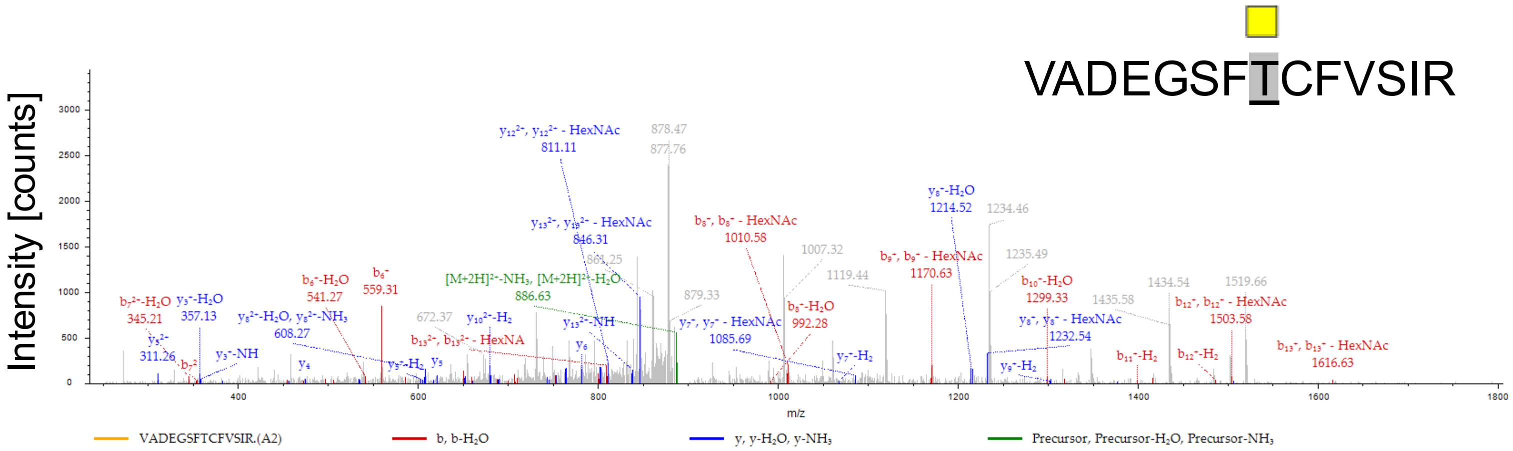

#1	b^{+}	b^{2+}	Seq.	y^{+}	y^{2+}	#2
1	100.07569	50.54148	V			14
2	171.11280	86.06004	A	1691.75813	846.38270	13
3	286.13975	143.57351	D	1620.72101	810.86415	12
4	415.18234	208.09481	E	1505.69407	753.35067	11
5	472.20380	236.60554	G	1376.65148	688.82938	10
6	559.23583	280.12155	S	1319.63001	660.31865	9
7	706.30425	353.65576	F	1232.59799	616.80263	8
8	1010.43130	505.71929	T-HexNAc	1085.52957	543.26842	7
9	1170.46195	585.73461	-Carbamidometh	781.40252	391.20490	6
10	1317.53036	659.26882	F	621.37187	311.18957	5
11	1416.59877	708.80303	V	474.30346	237.65537	4
12	1503.63080	752.31904	S	375.23504	188.12116	3
13	1616.71487	808.86107	I	288.20302	144.60515	2
14			R	175.11895	88.06311	1

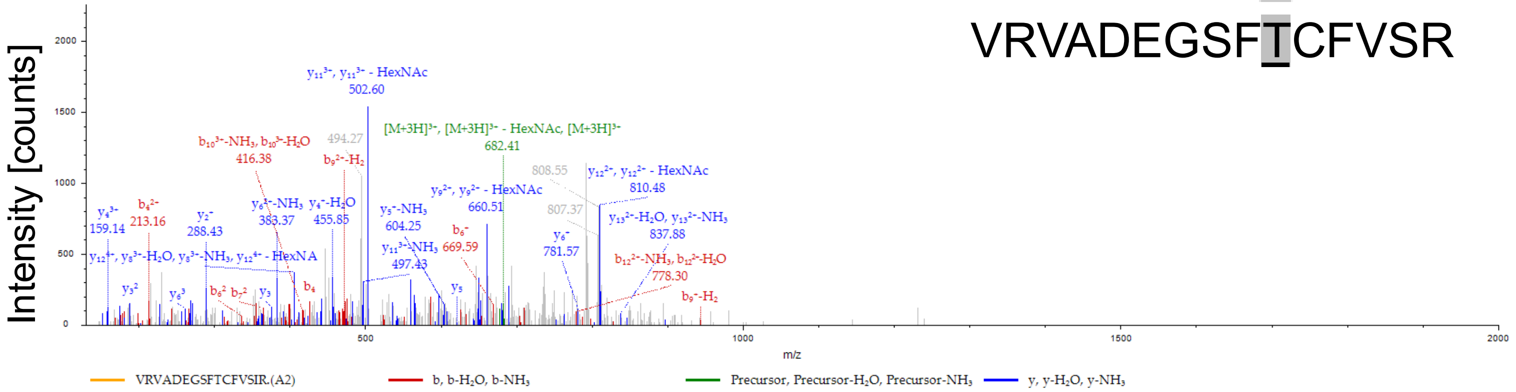

VRVADEGSFTCFVSR

#1	b^+	b^{2+}	b^{3+}	b^{4+}	Seq.	y^+	y^{2+}	y^{3+}	y^{4+}	#2
1	100.07569	50.54148	34.03008	25.77438	V					16
2	256.17680	128.59204	86.06378	64.79966	R	1946.92765	973.96746	649.64740	487.48737	15
3	355.24522	178.12625	119.08659	89.56676	V	1790.82654	895.91691	597.61370	448.46209	14
4	426.28233	213.64480	142.76563	107.32604	A	1691.75813	846.38270	564.59089	423.69499	13
5	541.30927	271.15827	181.10794	136.08278	D	1620.72101	810.86415	540.91186	405.93571	12
6	670.35187	335.67957	224.12214	168.34342	E	1505.69407	753.35067	502.56954	377.17898	11
7	727.37333	364.19030	243.12929	182.59879	G	1376.65148	688.82938	459.55534	344.91833	10
8	814.40536	407.70632	272.13997	204.35680	S	1319.63001	660.31865	440.54819	330.66296	9
9	961.47377	481.24052	321.16277	241.12390	F	1232.59799	616.80263	411.53751	308.90495	8
10	1265.60082	633.30405	422.53846	317.15566	T-HexNAc	1085.52957	543.26842	362.51471	272.13785	7
11	1425.63147	713.31937	475.88201	357.16333	-Carbamidometh	781.40252	391.20490	261.13902	196.10609	6
12	1572.69988	786.85358	524.90481	393.93043	F	621.37187	311.18957	207.79548	156.09843	5
13	1671.76830	836.38779	557.92762	418.69753	V	474.30346	237.65537	158.77267	119.33132	4
14	1758.80033	879.90380	586.93829	440.45554	S	375.23504	188.12116	125.74987	94.56422	3
15	1871.88439	936.44583	624.63298	468.72656	I	288.20302	144.60515	96.73919	72.80621	2
16					R	175.11895	88.06311	59.04450	44.53520	1

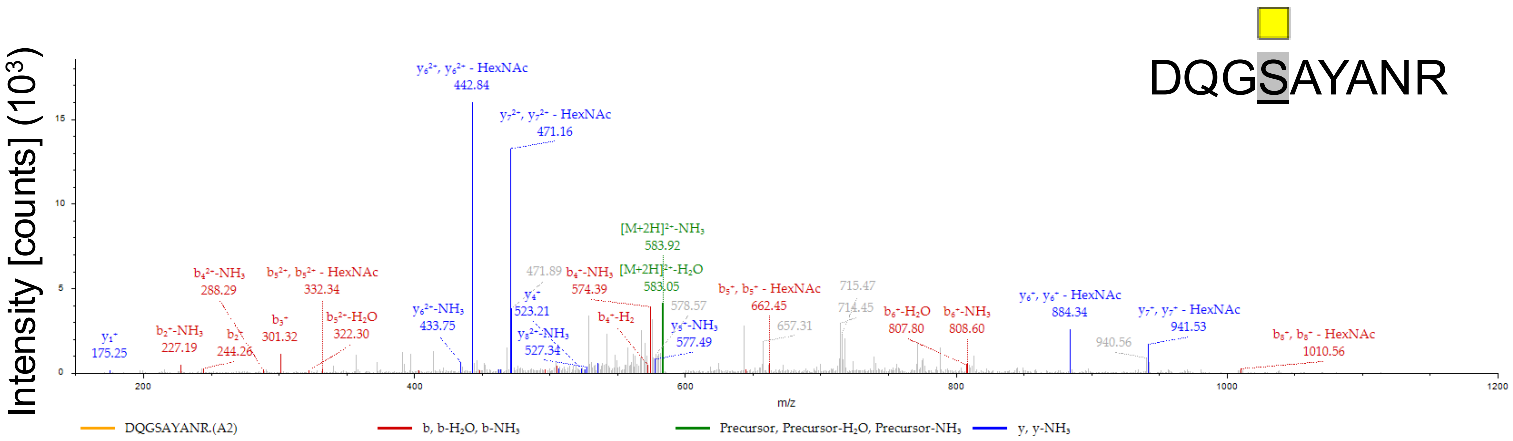

#1	b⁺	b²⁺	Seq.	y⁺	y²⁺	#2
1	116.03422	58.52075	D			9
2	244.09280	122.55004	Q	1069.49088	535.24908	8
3	301.11426	151.06077	G	941.43230	471.21979	7
4	591.22566	296.11647	S-HexNAc	884.41084	442.70906	6
5	662.26278	331.63503	A	594.29944	297.65336	5
6	825.32610	413.16669	Y	523.26232	262.13480	4
7	896.36322	448.68525	A	360.19899	180.60314	3
8	1010.40615	505.70671	N	289.16188	145.08458	2
9			R	175.11895	88.06311	1

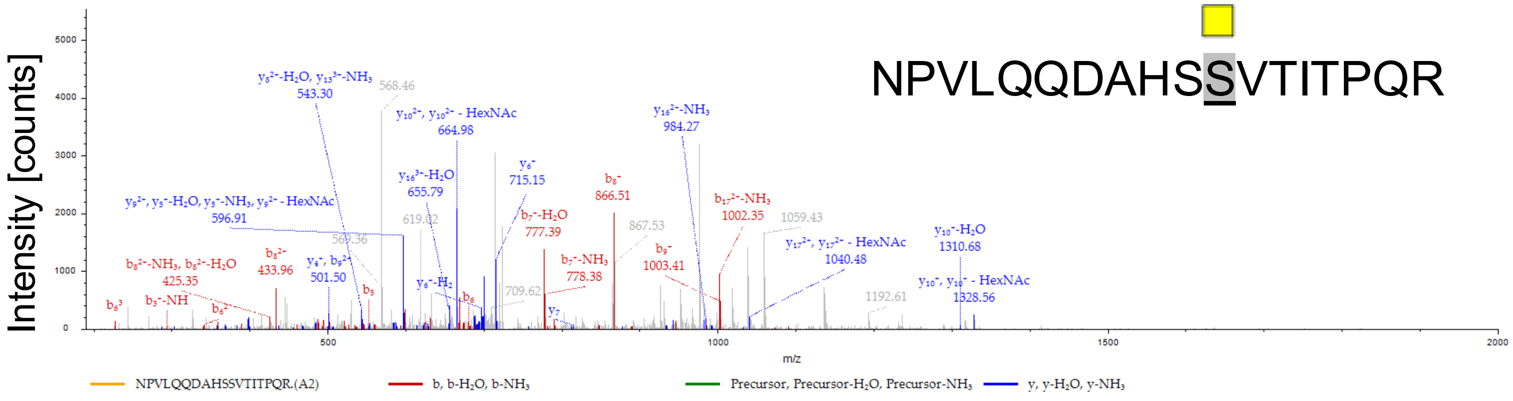

#1	b⁺	b²⁺	b³⁺	Seq.	y⁺	y²⁺	y³⁺	#2
1	115.05020	58.02874	39.02159	N				18
2	212.10297	106.55512	71.37251	P	2080.06692	1040.53710	694.02716	17
3	311.17138	156.08933	104.39531	V	1983.01416	992.01072	661.67624	16
4	424.25545	212.63136	142.09000	L	1883.94575	942.47651	628.65343	15
5	552.31402	276.66065	184.77619	Q	1770.86168	885.93448	590.95874	14
6	680.37260	340.68994	227.46238	Q	1642.80310	821.90519	548.27255	13
7	795.39954	398.20341	265.80470	D	1514.74453	757.87590	505.58636	12
8	866.43666	433.72197	289.48374	A	1399.71758	700.36243	467.24405	11
9	1003.49557	502.25142	335.17004	H	1328.68047	664.84387	443.56501	10
10	1090.52760	545.76744	364.18072	S	1191.62156	596.31442	397.87870	9
11	1380.63900	690.82314	460.88452	S-HexNAc	1104.58953	552.79840	368.86803	8
12	1479.70741	740.35734	493.90732	V	814.47813	407.74270	272.16423	7
13	1580.75509	790.88118	527.58988	T	715.40971	358.20850	239.14142	6
14	1693.83915	847.42322	565.28457	I	614.36204	307.68466	205.45886	5
15	1794.88683	897.94705	598.96713	T	501.27797	251.14262	167.76418	4
16	1891.93960	946.47344	631.31805	P	400.23029	200.61879	134.08162	3
17	2019.99817	1010.50273	674.00424	Q	303.17753	152.09240	101.73069	2
18				R	175.11895	88.06311	59.04450	1

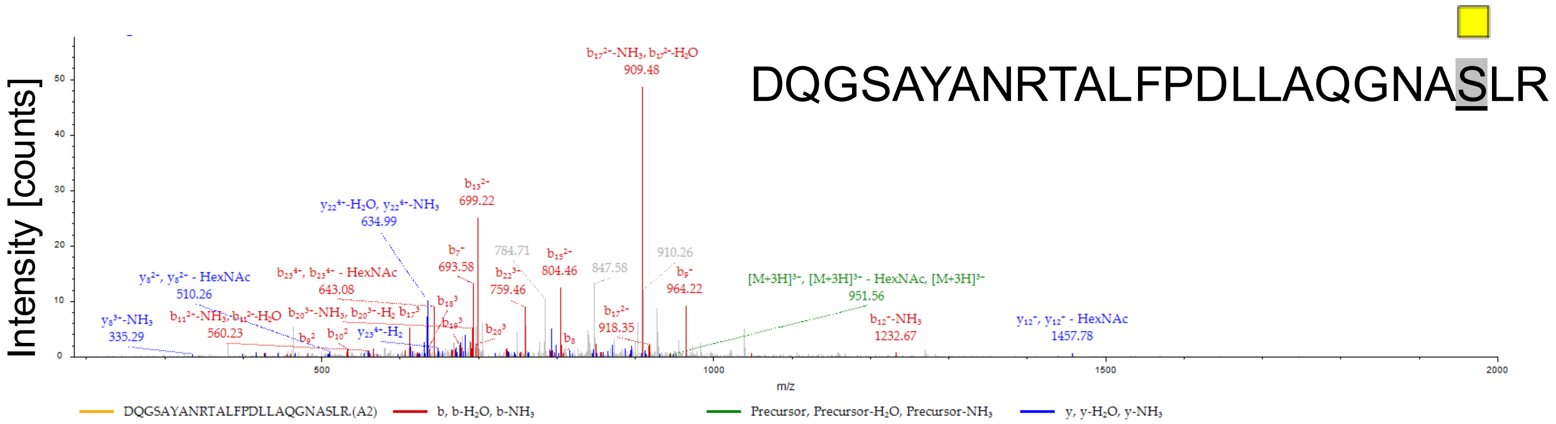

#1	b^{+}	b^{2+}	b^{3+}	b^{4+}	Seq.	y^{+}	y^{2+}	y^{3+}	y^{4+}	#2
1	116.03422	58.52075	39.34959	29.76401	D					25
2	244.09280	122.55004	82.03578	61.77866	Q	2738.37440	1369.69084	913.46298	685.34906	24
3	301.11426	151.06077	101.04294	76.03402	G	2610.31582	1305.66155	870.77679	653.33441	23
4	388.14629	194.57678	130.05361	97.79203	S	2553.29436	1277.15082	851.76964	639.07905	22
5	459.18340	230.09534	153.73265	115.55131	A	2466.26233	1233.63480	822.75896	617.32104	21
6	622.24673	311.62700	208.08709	156.31714	Y	2395.22521	1198.11625	799.07992	599.56176	20
7	693.28385	347.14556	231.76613	174.07642	A	2232.16189	1116.58458	744.72548	558.79593	19
8	808.31079	404.65903	270.10845	202.83315	N-Deamidated	2161.12477	1081.06602	721.04644	541.03665	18
9	964.41190	482.70959	322.14215	241.85843	R	2046.09783	1023.55255	682.70413	512.27991	17
10	1065.45958	533.23343	355.82471	267.12035	T	1889.99672	945.50200	630.67042	473.25464	16
11	1136.49669	568.75198	379.50375	284.87963	A	1788.94904	894.97816	596.98786	447.99272	15
12	1249.58076	625.29402	417.19844	313.15065	L	1717.91193	859.45960	573.30883	430.23344	14
13	1396.64917	698.82822	466.22124	349.91775	F	1604.82786	802.91757	535.61414	401.96242	13
14	1493.70193	747.35460	498.57216	374.18094	P	1457.75945	729.38336	486.59133	365.19532	12
15	1608.72888	804.86808	536.91448	402.93768	D	1360.70668	680.85698	454.24041	340.93213	11
16	1721.81294	861.41011	574.60916	431.20869	L	1245.67974	623.34351	415.89810	312.17539	10
17	1834.89700	917.95214	612.30385	459.47971	L	1132.59568	566.80148	378.20341	283.90438	9
18	1905.93412	953.47070	635.98289	477.23899	A	1019.51161	510.25945	340.50872	255.63336	8
19	2033.99270	1017.49999	678.66908	509.25363	Q	948.47450	474.74089	316.82968	237.87408	7
20	2091.01416	1046.01072	697.67624	523.50900	G	820.41592	410.71160	274.14349	205.85944	6
21	2205.05709	1103.03218	735.69055	552.01973	N	763.39446	382.20087	255.13634	191.60407	5
22	2276.09420	1138.55074	759.36958	569.77901	A	649.35153	325.17940	217.12203	163.09334	4
23	2566.20560	1283.60644	856.07338	642.30686	S-HexNAc	578.31442	289.66085	193.44299	145.33406	3
24	2679.28967	1340.14847	893.76807	670.57787	L	288.20302	144.60515	96.73919	72.80621	2
25					R	175.11895	88.06311	59.04450	44.53520	1

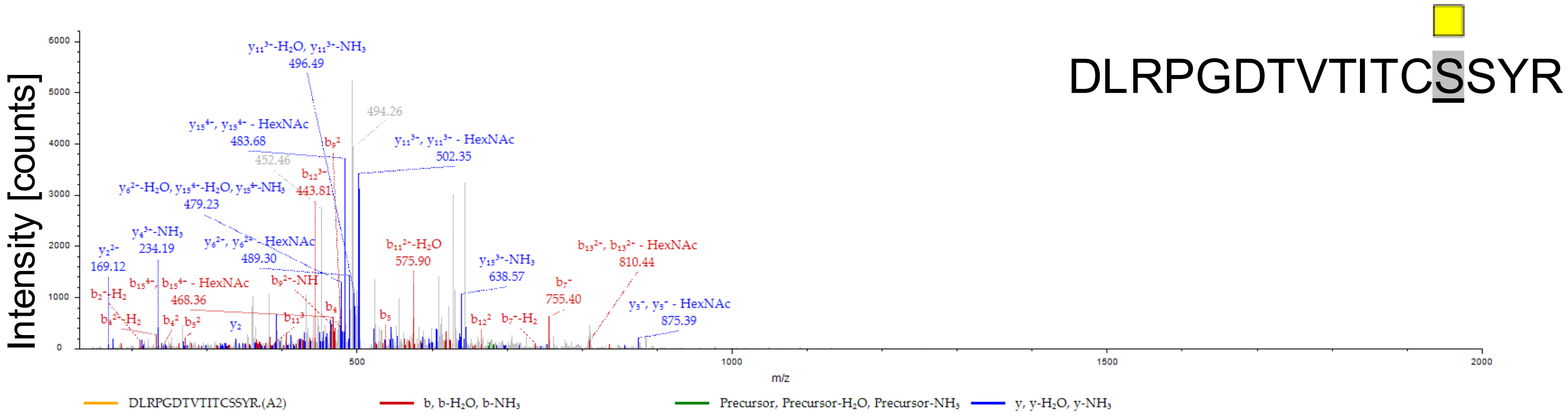

DLRPGDTVTITCSSYR

#1	b^{+}	b^{2+}	b^{3+}	Seq.	y^{+}	y^{2+}	y^{3+}	#2
1	116.03422	58.52075	39.34959	D				16
2	229.11828	115.06278	77.04428	L	1928.93822	964.97275	643.65092	15
3	385.21939	193.11334	129.07798	R	1815.85415	908.43071	605.95624	14
4	482.27216	241.63972	161.42890	P	1659.75304	830.38016	553.92253	13
5	539.29362	270.15045	180.43606	G	1562.70028	781.85378	521.57161	12
6	654.32056	327.66392	218.77837	D	1505.67881	753.34305	502.56446	11
7	755.36824	378.18776	252.46093	T	1390.65187	695.82957	464.22214	10
8	854.43666	427.72197	285.48374	V	1289.60419	645.30574	430.53958	9
9	955.48434	478.24581	319.16630	T	1190.53578	595.77153	397.51678	8
10	1068.56840	534.78784	356.86098	I	1089.48810	545.24769	363.83422	7
11	1169.61608	585.31168	390.54354	T	976.40404	488.70566	326.13953	6
12	1329.64673	665.32700	443.88709	-Carbamidometh	875.35636	438.18182	292.45697	5
13	1619.75813	810.38270	540.59089	S-HexNAc	715.32571	358.16649	239.11342	4
14	1706.79016	853.89872	569.60157	S	425.21431	213.11079	142.40962	3
15	1869.85348	935.43038	623.95601	Y	338.18228	169.59478	113.39894	2
16				R	175.11895	88.06311	59.04450	1

QLVHSFAEGQDQGSAYANR

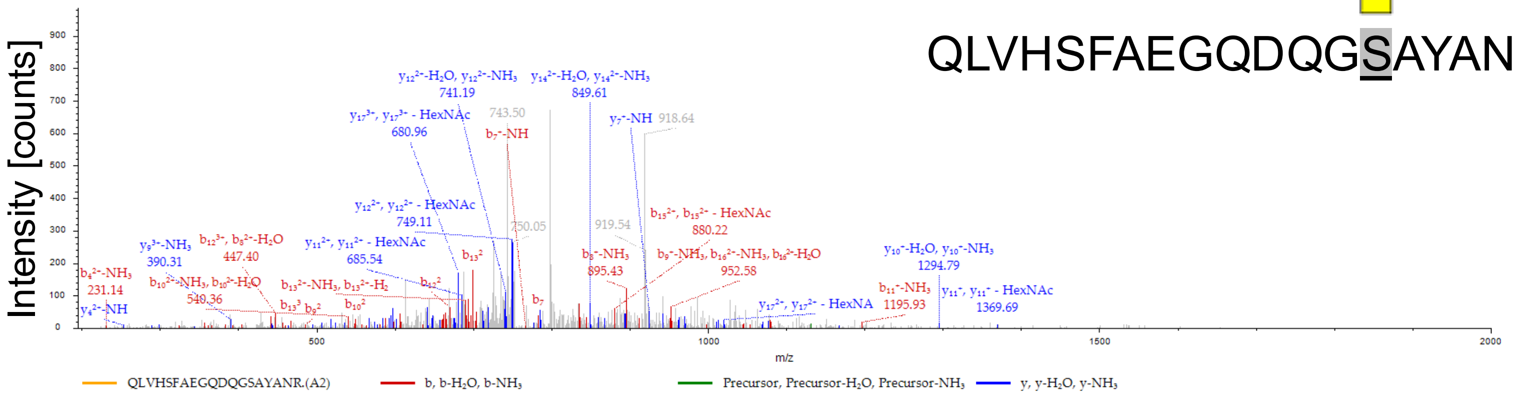

#1	b⁺	b²⁺	b³⁺	Seq.	y⁺	y²⁺	y³⁺	#2
1	129.06585	65.03657	43.69347	Q				19
2	242.14992	121.57860	81.38816	L	2152.98940	1076.99834	718.33465	18
3	341.21833	171.11280	114.41096	V	2039.90534	1020.45631	680.63996	17
4	478.27724	239.64226	160.09727	H	1940.83692	970.92210	647.61716	16
5	565.30927	283.15827	189.10794	S	1803.77801	902.39264	601.93085	15
6	712.37769	356.69248	238.13075	F	1716.74598	858.87663	572.92018	14
7	783.41480	392.21104	261.80978	A	1569.67757	785.34242	523.89737	13
8	912.45739	456.73233	304.82398	E	1498.64046	749.82387	500.21834	12
9	969.47886	485.24307	323.83114	G	1369.59786	685.30257	457.20414	11
10	1097.53743	549.27236	366.51733	Q	1312.57640	656.79184	438.19698	10
11	1212.56438	606.78583	404.85964	D	1184.51782	592.76255	395.51079	9
12	1340.62295	670.81512	447.54584	Q	1069.49088	535.24908	357.16848	8
13	1397.64442	699.32585	466.55299	G	941.43230	471.21979	314.48228	7
14	1687.75582	844.38155	563.25679	S-HexNAc	884.41084	442.70906	295.47513	6
15	1758.79293	879.90010	586.93583	A	594.29944	297.65336	198.77133	5
16	1921.85626	961.43177	641.29027	Y	523.26232	262.13480	175.09229	4
17	1992.89338	996.95033	664.96931	A	360.19899	180.60314	120.73785	3
18	2106.93630	1053.97179	702.98362	N	289.16188	145.08458	97.05881	2
19				R	175.11895	88.06311	59.04450	1

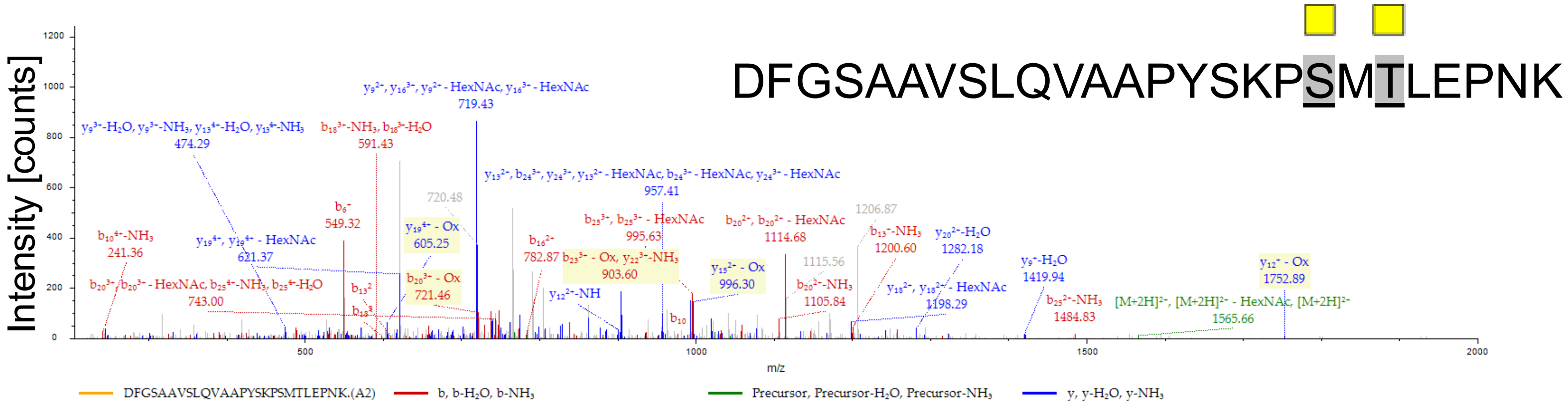

#1	b^{+}	b^{2+}	b^{3+}	b^{4+}	Seq.	y^{+}	y^{2+}	y^{3+}	y^{4+}	#2
1	116.03422	58.52075	39.34959	29.76401	D					26
2	263.10263	132.05496	88.37240	66.53112	F	3015.48671	1508.24699	1005.83375	754.62714	25
3	320.12410	160.56569	107.37955	80.78648	G	2868.41830	1434.71279	956.81095	717.86003	24
4	407.15613	204.08170	136.39023	102.54449	S	2811.39683	1406.20205	937.80380	703.60467	23
5	478.19324	239.60026	160.06926	120.30377	A	2724.36480	1362.68604	908.79312	681.84666	22
6	549.23035	275.11881	183.74830	138.06305	A	2653.32769	1327.16748	885.11408	664.08738	21
7	648.29877	324.65302	216.77111	162.83015	V	2582.29058	1291.64893	861.43504	646.32810	20
8	735.33080	368.16904	245.78178	184.58816	S	2483.22216	1242.11472	828.41224	621.56100	19
9	848.41486	424.71107	283.47647	212.85917	L	2396.19013	1198.59871	799.40156	599.80299	18
10	976.47344	488.74036	326.16266	244.87382	Q	2283.10607	1142.05667	761.70687	571.53198	17
11	1075.54185	538.27456	359.18547	269.64092	V	2155.04749	1078.02739	719.02068	539.51733	16
12	1146.57896	573.79312	382.86451	287.40020	A	2055.97908	1028.49318	685.99788	514.75023	15
13	1217.61608	609.31168	406.54354	305.15948	A	1984.94197	992.97462	662.31884	496.99095	14
14	1314.66884	657.83806	438.89447	329.42267	P	1913.90485	957.45606	638.63980	479.23167	13
15	1477.73217	739.36972	493.24891	370.18850	Y	1816.85209	908.92968	606.28888	454.96848	12
16	1564.76420	782.88574	522.25958	391.94651	S	1653.78876	827.39802	551.93444	414.20265	11
17	1692.85916	846.93322	564.95790	423.97025	K	1566.75673	783.88200	522.92376	392.44464	10
18	1789.91193	895.45960	597.30883	448.23344	P	1438.66177	719.83452	480.22544	360.42090	9
19	2080.02333	1040.51530	694.01263	520.76129	S-HexNAc	1341.60900	671.30814	447.87452	336.15771	8
20	2227.05873	1114.03300	743.02443	557.52014	M-Oxidation	1051.49760	526.25244	351.17072	263.62986	7
21	2531.18578	1266.09653	844.40011	633.55190	T-HexNAc	904.46220	452.73474	302.15892	226.87101	6
22	2644.26984	1322.63856	882.09480	661.82292	L	600.33515	300.67121	200.78324	150.83925	5
23	2773.31243	1387.15986	925.10900	694.08357	E	487.25109	244.12918	163.08855	122.56823	4
24	2870.36520	1435.68624	957.45992	718.34676	P	358.20850	179.60789	120.07435	90.30758	3
25	2984.40813	1492.70770	995.47423	746.85749	N	261.15573	131.08150	87.72343	66.04439	2
26					K	147.11280	74.06004	49.70912	37.53366	1

o

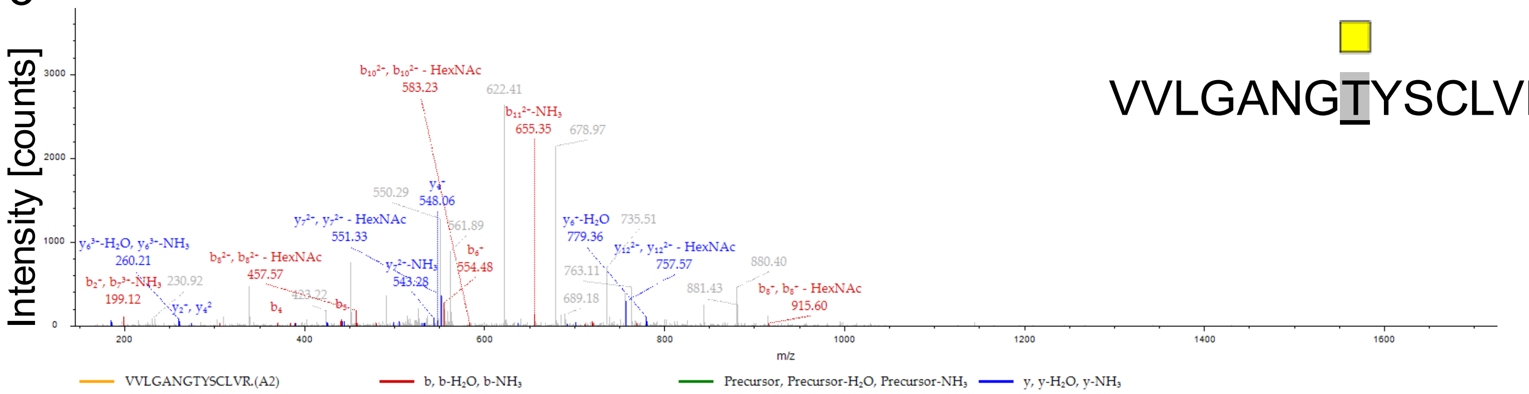

VVLGANGTYSCLVR

#1	b⁺	b²⁺	b³⁺	Seq.	y⁺	y²⁺	y³⁺	#2
1	100.07569	50.54148	34.03008	V				14
2	199.14410	100.07569	67.05289	V	1612.79993	806.90360	538.27150	13
3	312.22817	156.61772	104.74757	L	1513.73152	757.36940	505.24869	12
4	369.24963	185.12845	123.75473	G	1400.64746	700.82737	467.55400	11
5	440.28675	220.64701	147.43377	A	1343.62599	672.31663	448.54685	10
6	554.32967	277.66847	185.44808	N	1272.58888	636.79808	424.86781	9
7	611.35114	306.17921	204.45523	G	1158.54595	579.77661	386.85350	8
8	915.47819	458.24273	305.83091	T-HexNAc	1101.52449	551.26588	367.84635	7
9	1078.54152	539.77440	360.18536	Y	797.39744	399.20236	266.47066	6
10	1165.57355	583.29041	389.19603	S	634.33411	317.67069	212.11622	5
11	1325.60419	663.30574	442.53958	-Carbamidometh	547.30208	274.15468	183.10554	4
12	1438.68826	719.84777	480.23427	L	387.27143	194.13935	129.76199	3
13	1537.75667	769.38197	513.25707	V	274.18737	137.59732	92.06731	2
14				R	175.11895	88.06311	59.04450	1

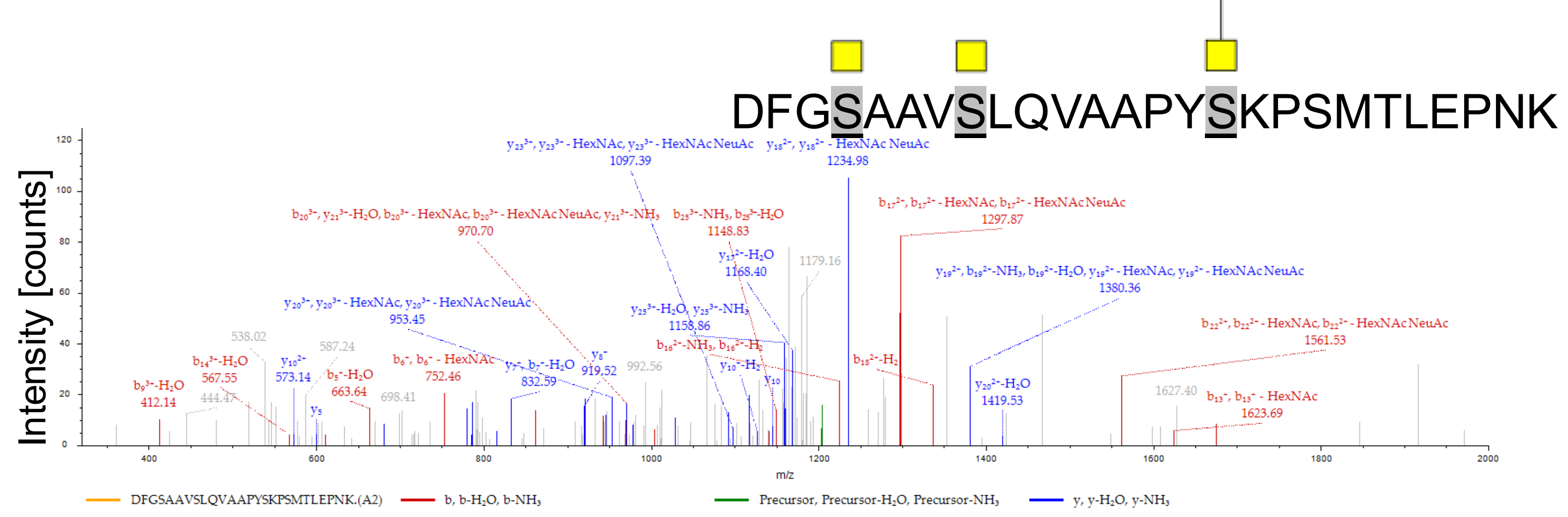

#1	b^{+}	b^{2+}	b^{3+}	Seq.	y^{+}	y^{2+}	y^{3+}	#2
1	116.03422	58.52075	39.34959	D				26
2	263.10263	132.05496	88.37240	F	3493.66658	1747.33693	1165.22705	25
3	320.12410	160.56569	107.37955	G	3346.59817	1673.80272	1116.20424	24
4	610.23550	305.62139	204.08335	S-HexNAc	3289.57671	1645.29199	1097.19709	23
5	681.27261	341.13994	227.76239	A	2999.46531	1500.23629	1000.49329	22
6	752.30973	376.65850	251.44143	A	2928.42819	1464.71773	976.81425	21
7	851.37814	426.19271	284.46423	V	2857.39108	1429.19918	953.13521	20
8	1141.48954	571.24841	381.16803	S-HexNAc	2758.32266	1379.66497	920.11241	19
9	1254.57361	627.79044	418.86272	L	2468.21126	1234.60927	823.40861	18
10	1382.63218	691.81973	461.54891	Q	2355.12720	1178.06724	785.71392	17
11	1481.70060	741.35394	494.57172	V	2227.06862	1114.03795	743.02772	16
12	1552.73771	776.87249	518.25075	A	2128.00021	1064.50374	710.00492	15
13	1623.77482	812.39105	541.92979	A	2056.96309	1028.98519	686.32588	14
14	1720.82759	860.91743	574.28071	P	1985.92598	993.46663	662.64684	13
15	1883.89092	942.44910	628.63516	Y	1888.87322	944.94025	630.29592	12
16	2465.09773	1233.05251	822.37076	HexNAc(1)NeuAc	1725.80989	863.40858	575.94148	11
17	2593.19270	1297.09999	865.06908	K	1144.60307	572.80517	382.20587	10
18	2690.24546	1345.62637	897.42000	P	1016.50811	508.75769	339.50755	9
19	2777.27749	1389.14238	926.43068	S	919.45534	460.23131	307.15663	8
20	2908.31797	1454.66262	970.11084	M	832.42332	416.71530	278.14596	7
21	3009.36565	1505.18646	1003.79340	T	701.38283	351.19505	234.46579	6
22	3122.44972	1561.72850	1041.48809	L	600.33515	300.67121	200.78324	5
23	3251.49231	1626.24979	1084.50229	E	487.25109	244.12918	163.08855	4
24	3348.54507	1674.77617	1116.85321	P	358.20850	179.60789	120.07435	3
25	3462.58800	1731.79764	1154.86752	N	261.15573	131.08150	87.72343	2
26				K	147.11280	74.06004	49.70912	1

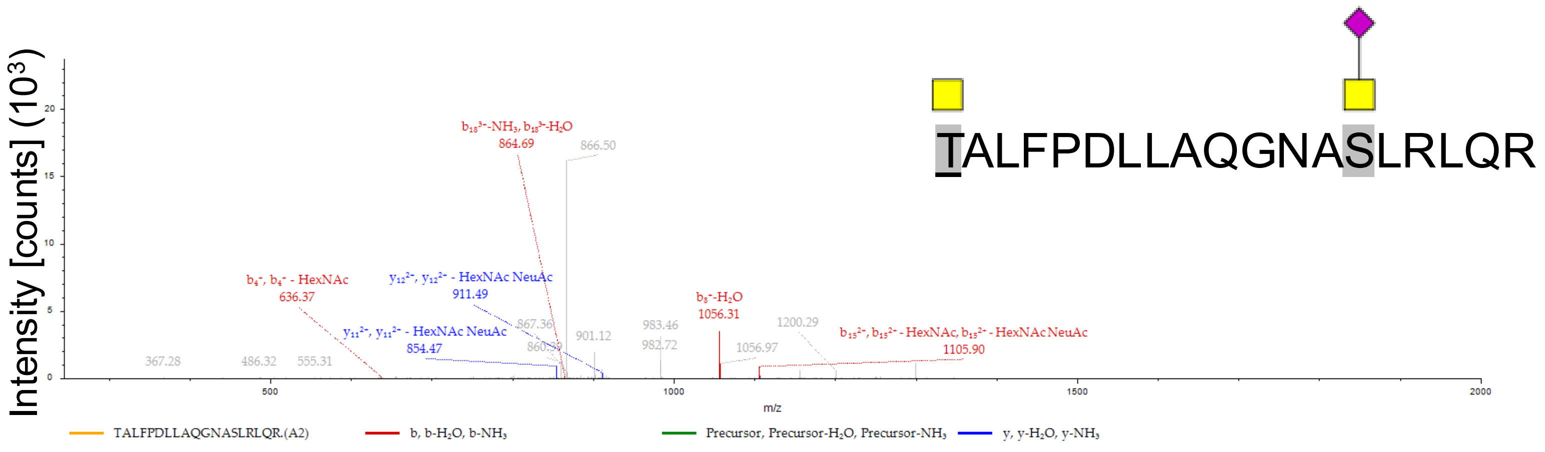

#1	b^{+}	b^{2+}	b^{3+}	Seq.	y^{+}	y^{2+}	y^{3+}	#2
1	305.13433	153.07080	102.38296	T-HexNAc				19
2	376.17144	188.58936	126.06200	A	2478.27222	1239.63975	826.76226	18
3	489.25551	245.13139	163.75669	L	2407.23511	1204.12119	803.08322	17
4	636.32392	318.66560	212.77949	F	2294.15105	1147.57916	765.38853	16
5	733.37668	367.19198	245.13041	P	2147.08263	1074.04495	716.36573	15
6	848.40363	424.70545	283.47273	D	2050.02987	1025.51857	684.01481	14
7	961.48769	481.24748	321.16741	L	1935.00293	968.00510	645.67249	13
8	1074.57175	537.78952	358.86210	L	1821.91886	911.46307	607.97781	12
9	1145.60887	573.30807	382.54114	A	1708.83480	854.92104	570.28312	11
10	1273.66745	637.33736	425.22733	Q	1637.79768	819.40248	546.60408	10
11	1330.68891	665.84809	444.23449	G	1509.73911	755.37319	503.91789	9
12	1445.71585	723.36156	482.57680	N-Deamidated	1452.71764	726.86246	484.91073	8
13	1516.75297	758.88012	506.25584	A	1337.69070	669.34899	446.56842	7
14	2097.95978	1049.48353	699.99145	HexNAc(1)NeuAc	1266.65359	633.83043	422.88938	6
15	2211.04385	1106.02556	737.68613	L	685.44677	343.22702	229.15377	5
16	2367.14496	1184.07612	789.71984	R	572.36270	286.68499	191.45909	4
17	2480.22902	1240.61815	827.41453	L	416.26159	208.63444	139.42538	3
18	2608.28760	1304.64744	870.10072	Q	303.17753	152.09240	101.73069	2
19				R	175.11895	88.06311	59.04450	1

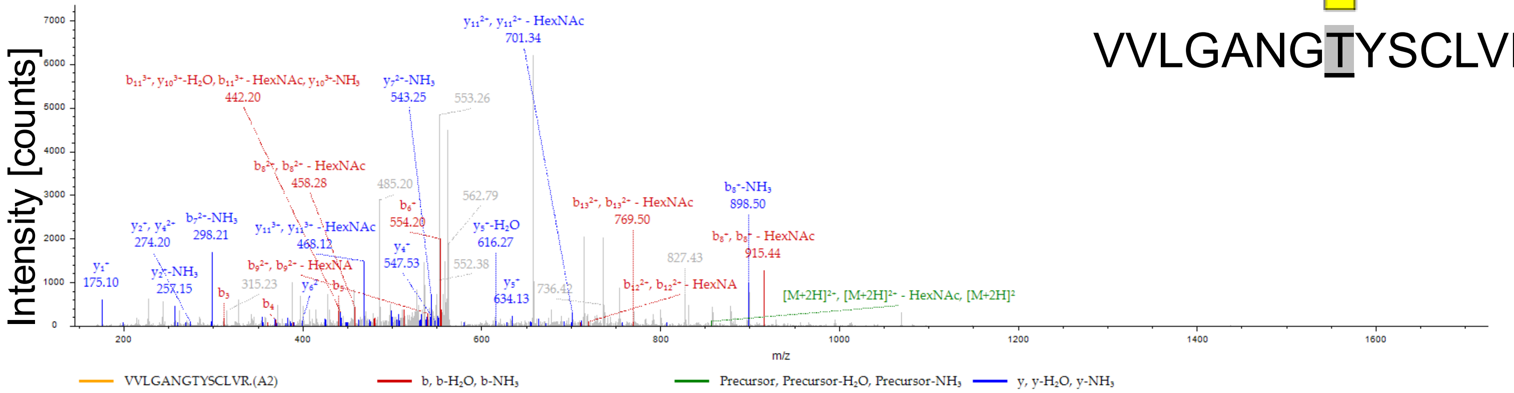

VVLGANGTYSCLVR

#1	b⁺	b²⁺	b³⁺	Seq.	y⁺	y²⁺	y³⁺	#2
1	100.07569	50.54148	34.03008	V				14
2	199.14410	100.07569	67.05289	V	1612.79993	806.90360	538.27150	13
3	312.22817	156.61772	104.74757	L	1513.73152	757.36940	505.24869	12
4	369.24963	185.12845	123.75473	G	1400.64746	700.82737	467.55400	11
5	440.28675	220.64701	147.43377	A	1343.62599	672.31663	448.54685	10
6	554.32967	277.66847	185.44808	N	1272.58888	636.79808	424.86781	9
7	611.35114	306.17921	204.45523	G	1158.54595	579.77661	386.85350	8
8	915.47819	458.24273	305.83091	T-HexNAc	1101.52449	551.26588	367.84635	7
9	1078.54152	539.77440	360.18536	Y	797.39744	399.20236	266.47066	6
10	1165.57355	583.29041	389.19603	S	634.33411	317.67069	212.11622	5
11	1325.60419	663.30574	442.53958	-Carbamidometh	547.30208	274.15468	183.10554	4
12	1438.68826	719.84777	480.23427	L	387.27143	194.13935	129.76199	3
13	1537.75667	769.38197	513.25707	V	274.18737	137.59732	92.06731	2
14				R	175.11895	88.06311	59.04450	1

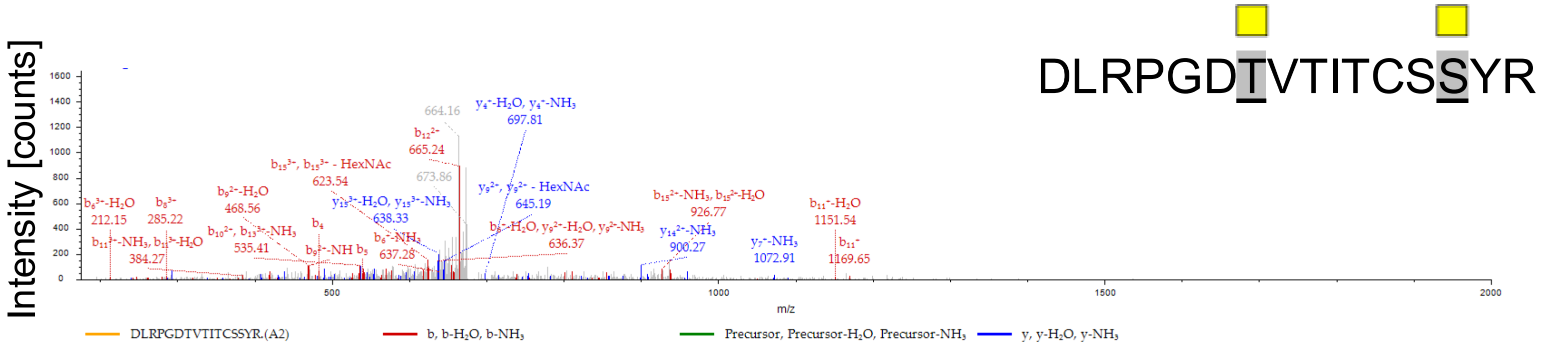

#1	b⁺	b²⁺	b³⁺	Seq.	y⁺	y²⁺	y³⁺	#2
1	116.03422	58.52075	39.34959	D				16
2	229.11828	115.06278	77.04428	L	2132.01759	1066.51243	711.34405	15
3	385.21939	193.11334	129.07798	R	2018.93353	1009.97040	673.64936	14
4	482.27216	241.63972	161.42890	P	1862.83242	931.91985	621.61566	13
5	539.29362	270.15045	180.43606	G	1765.77965	883.39346	589.26473	12
6	654.32056	327.66392	218.77837	D	1708.75819	854.88273	570.25758	11
7	958.44762	479.72745	320.15406	T-HexNAc	1593.73124	797.36926	531.91527	10
8	1057.51603	529.26165	353.17686	V	1289.60419	645.30574	430.53958	9
9	1158.56371	579.78549	386.85942	T	1190.53578	595.77153	397.51678	8
10	1271.64777	636.32752	424.55411	I	1089.48810	545.24769	363.83422	7
11	1372.69545	686.85136	458.23667	T	976.40404	488.70566	326.13953	6
12	1532.72610	766.86669	511.58022	-Carbamidometh	875.35636	438.18182	292.45697	5
13	1619.75813	810.38270	540.59089	S	715.32571	358.16649	239.11342	4
14	1909.86953	955.43840	637.29469	S-HexNAc	628.29368	314.65048	210.10275	3
15	2072.93286	1036.97007	691.64914	Y	338.18228	169.59478	113.39894	2
16				R	175.11895	88.06311	59.04450	1
